# *Dioscorea oppositifolia* L. Attenuates Weaning-Induced Intestinal Injury by Regulating Oxidative Stress and Apoptosis in Piglets

**DOI:** 10.3390/vetsci13040365

**Published:** 2026-04-08

**Authors:** Xiongwei Shi, Shaoguang Ge, Haimin Wang, Xiaowang Chen, Xiangyi Pan, Chen Liu, Zhengying Qiu, Wenshu Zou, Hao Cao, Yujia Liu, Qiyu Bai, Ruihua Xin

**Affiliations:** 1 School of Pharmacy, Gansu University of Chinese Medicine, Lanzhou 730101, China; 2 Lanzhou Institute of Husbandry and Pharmaceutical Sciences, Engineering and Technology Research Center of Traditional Chinese Veterinary Medicine of Gansu Province, Key Laboratory of Veterinary Pharmaceutical Development of Ministry of Agriculture and Rural Affairs of P.R. Lanzhou, Chinese Academy of Agricultural Sciences (CAAS), Lanzhou 730050, Chinah77113630@gmail.com (H.C.);; 3 Institute of Traditional Chinese Medicine Health Industry, China Academy of Chinese Medical Sciences, Nanchang 330115, China

**Keywords:** Chinese yam, weaned piglets, intestinal injury, oxidative stress, apoptosis

## Abstract

Weaning stress is frequently associated with intestinal oxidative stress, inflammatory activation, and epithelial apoptosis in piglets. This study investigated whether dietary supplementation with Chinese yam (*Dioscorea oppositifolia* L., YAM) alleviates weaning-induced intestinal injury by modulating the oxidative stress-inflammation-apoptosis axis. 48 weaned piglets were assigned to a control diet or diets supplemented with low (1%)/high (2%) doses of YAM. Intestinal morphology, antioxidant capacity, inflammatory signaling, and apoptosis-related markers were assessed, and jejunal transcriptomic profiling was also performed. Supplementing with YAM improved villus architecture and enhanced intestinal antioxidant properties, manifested as increased total antioxidant capacity and reduced malondialdehyde levels. At the molecular level, YAM activated the Keap-1/Nrf2/HO-1 pathway and upregulated the expression of antioxidant-related genes, including superoxide dismutase 2 (SOD2), catalase (CAT), and NAD(P)H quinone dehydrogenase 1 (NQO1), and suppressed NF-κB signaling by reducing Myd88 and p-p65 protein levels. In addition, YAM modulated mitochondrial apoptosis by upregulating Bcl-2 and reducing the expression of Bax and Cleaved caspase-3. Transcriptomic analysis identified 1227 differentially expressed genes between the control and high-dose groups (784 upregulated and 443 downregulated). Mechanism-oriented module analysis further confirmed coordinated enhancement of antioxidant pathways alongside suppression of inflammatory and apoptotic gene signatures. These findings demonstrate that dietary YAM supplementation attenuates weaning-associated intestinal injury by rebalancing oxidative stress, inflammatory signaling, and apoptosis-related pathways, thereby supporting its potential application as a functional feed additive in swine production.

## Simple Summary

Weaning stress often leads to intestinal damage, oxidative imbalance, and impaired growth in piglets. Natural plant-derived feed additives are increasingly explored as alternatives to antibiotics for maintaining intestinal health during this critical period. Chinese yam (*Dioscorea oppositifolia* L., YAM) is a traditional edible plant rich in bioactive compounds with antioxidant and anti-inflammatory properties. In this study, dietary supplementation with YAM was evaluated for its ability to alleviate intestinal injury in weaned piglets. The results showed that YAM improved intestinal morphology, enhanced antioxidant capacity, reduced inflammatory responses, and inhibited excessive epithelial apoptosis. Transcriptomic analysis further revealed coordinated changes in genes related to oxidative stress, inflammation, and apoptosis. These findings suggest that dietary YAM may help improve intestinal health and resilience to weaning stress in piglets, supporting its use as a functional feed additive in pig production.

## 1. Introduction

Weaning represents a critical transitional stage in pig production and is frequently associated with increased susceptibility to enteric disorders [1,2]. Abrupt changes in diet and environment disrupt intestinal barrier integrity, impair nutrient absorption, and compromise growth performance [3,4]. Therefore, maintaining intestinal homeostasis during the weaning period remains a major concern in veterinary practice and swine production [5].

Oxidative stress plays a central role in weaning-associated intestinal injury [6,7]. Excessive production of reactive oxygen species (ROS) disrupts redox homeostasis, leading to lipid peroxidation and depletion of endogenous antioxidant defenses [8]. The antioxidant defense system, including enzymes such as superoxide dismutase (SOD), catalase (CAT), and Nrf2-regulated enzymes, including heme oxygenase-1 (HO-1) and NADH quinone oxidoreductase 1 (NQO1), is essential for maintaining redox homeostasis. Impairment of these antioxidant mechanisms can amplify oxidative damage and weaken epithelial integrity [9,10]. Oxidative stress is closely linked to inflammatory activation [11,12]. Accumulated ROS can stimulate NF-κB signaling, thereby promoting the expression of pro-inflammatory mediators such as inducible nitric oxide synthase (iNOS/NOS2) and interleukin-1β (IL-1β) [13,14,15]. Sustained inflammatory responses further aggravate epithelial injury and disrupt mucosal barrier function [16]. Meanwhile, oxidative and inflammatory stress may converge on mitochondrial pathways [17], facilitating the activation of pro-apoptotic regulators such as Bax and caspases while reducing anti-apoptotic proteins, including Bcl-2, ultimately contributing to epithelial cell apoptosis and mucosal damage [18,19,20]. Regulation of this oxidative stress-inflammation-apoptosis axis is therefore considered central to preserving intestinal integrity during weaning [21].

With increasing restrictions on the use of in-feed antibiotics in animal production, natural dietary supplements have attracted growing attention as potential alternatives for maintaining intestinal health [22,23,24,25]. *Dioscorea oppositifolia* L. (Chinese yam, YAM) is a traditional edible plant rich in bioactive constituents, including polysaccharides, steroidal saponins, polyphenols, and flavonoids [26,27,28,29]. Previous studies have indicated that YAM supplementation is associated with enhanced antioxidant capacity and improved mucosal defense [30,31,32]. Among these components, YAM polysaccharides are considered a major contributor to its antioxidant activity [32]. It has also been shown that crude extracts of YAM can protect intestinal epithelial cells from H_2_O_2_-induced oxidative injury by enhancing antioxidant defenses and inhibiting NF-κB signaling. In addition, anthocyanins derived from YAM have been reported to improve antioxidant capacity and immune function in Hainan black goats [33,34]. Steroidal saponins derived from YAM have been reported to exhibit anti-inflammatory activity by suppressing NF-κB signaling and decreasing the production of pro-inflammatory cytokines, including TNF-α and IL-6 [35]. YAM-derived polysaccharides exhibit antioxidant activity by reducing ROS and malondialdehyde (MDA) levels and enhancing SOD activity. YAM extracts also exert anti-inflammatory effects via MAPK and NF-κB signaling pathways [36,37]. Phenolic compounds isolated from YAM have been reported to attenuate intestinal mucosal injury by suppressing NF-κB/COX-2 signaling and reducing epithelial apoptosis. YAM polysaccharides have been shown to inhibit oxidative stress-induced programmed cell death in macrophages [38,39], suggesting their potential involvement in the modulation of mitochondrial-dependent apoptotic pathways.

In our previous work, dietary supplementation with YAM in weaned piglets under production conditions demonstrated good safety and functional efficacy, as evidenced by improved growth performance, reduced diarrhea incidence, increased serum immunoglobulin levels, enhanced expression of intestinal tight junction proteins and improved gut microbiota composition [40]. These findings provide a practical basis for the application of YAM as a functional feed additive during the weaning period. However, whether YAM exerts its protective effects under weaning stress through coordinated regulation of the intestinal oxidative stress-inflammation-apoptosis axis, and the underlying molecular mechanisms, remain to be elucidated.

Therefore, the present study aimed to investigate the effects of dietary YAM supplementation on intestinal oxidative stress, inflammatory signaling pathways, and apoptosis in weaned piglets. By integrating molecular biology approaches with transcriptomic analysis, we aimed to further elucidate the mechanisms underlying its intestinal protective effects during the weaning period.

## 2. Materials and Methods

### 2.1. YAM Powder Processing and Composition Analysis

YAM was purchased from Zhengde Tang Pharmaceutical Co., Ltd. (Lanzhou, China). The raw materials were air-dried at room temperature, ground into powder, and sieved through a 25-mesh screen. The prepared powder was divided into portions, sealed, and stored in a cool, dry environment until use. YAM powder was incorporated into the experimental diets according to the predetermined supplementation levels. The main nutrient composition of YAM was analyzed according to the following Chinese national standard methods: GB/T 6434-2022, GB/T 6437-2018, GB/T 6436-2018, GB/T 6438-2007, GB/T 6433-2006, GB/T 20806-2022, GB/T 6435-2014, and GB/T 6432-2018. These standards are publicly available through the National Standards Full-Text Public Service Platform (https://openstd.samr.gov.cn). Composition analysis indicated that YAM consisted of 90.92% neutral detergent fiber, 4.60% crude fiber, 1.70% crude ash, 0.60% crude fat, 0.243% total calcium, 0.11% total phosphorus, 7.44% moisture, and 8.55% crude protein.

### 2.2. Experimental Design and Animal Management

A total of 48 healthy weaned piglets (PIC three-way crossbred; initial body weight 11.39 ± 0.07 kg) were obtained from Sichuan Guotou Qiangshan Science and Technology Group Co., Ltd. (Mian Yang, China). The animals were randomly assigned to three dietary treatments (*n* = 16 per group) and maintained under identical management conditions. Piglets in the control group were fed a basal diet, whereas those in the treatment groups received the basal diet supplemented with 1% YAM powder (YAML) or 2% YAM powder (YAMH); the doses were selected based on preliminary trials. The experimental period lasted for 28 days. Feed and water were provided ad libitum throughout the experiment. The housing facility was maintained at an ambient temperature of 28 ± 2 °C with relative humidity controlled at 65 ± 5%. The formulation and nutrient composition of the basal diet were identical to those described in our previous study [40] and are provided in Supplementary Table S1.

### 2.3. Ethical Approval

All experimental procedures involving animals were reviewed and approved by the Animal Ethics Committee of the Lanzhou Institute of Husbandry and Pharmaceutical Sciences, Chinese Academy of Agricultural Sciences (Approval No. 2023-021), and were carried out in accordance with relevant national and institutional guidelines.

### 2.4. Sample Collection

At the end of the 28-day experimental period, piglets were fasted for 12 h with free access to water before sample collection. Blood samples were obtained from the anterior vena cava and centrifuged at 3000× *g* for 10 min at 4 °C to separate serum, which was then stored at −80 °C for subsequent antioxidant analysis. Following blood collection, piglets were euthanized by intravenous administration of sodium pentobarbital. Intestinal segments (duodenum, jejunum, and ileum) were rapidly excised. After rinsing with ice-cold physiological saline, tissues were snap-frozen in liquid nitrogen and stored at −80 °C for further biochemical and molecular analyses. Additional samples were fixed in 4% paraformaldehyde for histological and immunohistochemical evaluation.

### 2.5. Serum Antioxidant Enzyme Assays

Serum antioxidant parameters were determined using commercial assay kits in accordance with the manufacturers’ instructions. Superoxide dismutase (SOD) activity was measured using a kit purchased from Nanjing Jiancheng Bioengineering Institute (Nanjing, China; No. A001-3). Total antioxidant capacity (T-AOC; No. BC1315), MDA (No. BC0025), and CAT (No. BC0205) levels were determined using assay kits obtained from Beijing Solarbio Science & Technology Co., Ltd. (Beijing, China). All indices were analyzed using colorimetric methods, and absorbance values were measured with a microplate reader according to the respective assay protocols.

### 2.6. Histological Analysis

Intestinal samples from the duodenum, jejunum, and ileum were fixed in 4% paraformaldehyde, dehydrated using a graded ethanol series, embedded in paraffin, and sectioned into 5 μm slices. The sections were then stained with hematoxylin and eosin (H&E) for histological assessment. Histological evaluation was performed under a light microscope (Olympus, Tokyo, Japan) to assess intestinal mucosal morphology. Structural features including villus integrity, epithelial continuity, villus arrangement, and the presence of epithelial shedding or mucosal damage were qualitatively evaluated.

### 2.7. Immunohistochemical Analysis

Paraffin-embedded jejunal sections were deparaffinized, rehydrated, and subjected to antigen retrieval in citrate buffer (pH 6.0). Endogenous peroxidase activity was blocked with 3% hydrogen peroxide for 10 min at room temperature. After blocking with 5% normal goat serum for 30 min, sections were incubated with a rabbit anti-Bax polyclonal antibody (1:200, Cat No. 60267, Proteintech, Rosemont, IL, USA) overnight at 4 °C. After washing, sections were incubated with a horseradish peroxidase (HRP)-conjugated goat anti-rabbit secondary antibody (1:500, Cat No. 81290, Proteintech, USA) for 1 h at room temperature. Immunoreactive signals were visualized using 3,3′-diaminobenzidine (DAB) as the chromogen, and nuclei were counterstained with hematoxylin. For quantitative analysis, five randomly selected fields per section were captured using a light microscope (DM4000B, Leica, Wetzlar, Germany) at 200× magnification. The integrated optical density (IOD) of Bax-positive staining was measured using ImageJ software (version 1.53 NIH, Bethesda, MD, USA), and the mean IOD value per field was calculated for statistical comparison.

### 2.8. Western Blot Analysis

Intestinal tissues were homogenized in lysis buffer containing protease and phosphatase inhibitors. The homogenates were centrifuged at 12,000× *g* at 4 °C, and the resulting supernatants were collected for protein extraction. Protein concentrations were measured using a BCA protein assay kit (P0012S Beyotime Biotechnology, Shanghai, China). Equal amounts of protein were denatured, separated by SDS-PAGE, and transferred onto nitrocellulose membranes. After blocking with 5% non-fat milk, the membranes were incubated with primary antibodies at 4 °C overnight, followed by incubation with corresponding secondary antibodies. Protein bands were visualized using enhanced chemiluminescence (ECL) reagents (Thermo Fisher Scientific, Waltham, MA, USA) and quantified by densitometry using ImageJ software. Band intensities were measured by calculating the integrated density, and target protein expression levels were normalized to β-actin. Primary antibodies against Nrf2, Keap-1, HO-1, Bax, Bcl-2, and Cleaved caspase-3 were applied at a dilution of 1:1000 (Proteintech, USA).

### 2.9. RT-qPCR Analysis

Total RNA was isolated from intestinal tissues stored at −80 °C using TRIzol reagent (Invitrogen, Carlsbad, CA, USA) in accordance with the manufacturer’s instructions. RNA concentration and purity were assessed using a spectrophotometer. cDNA was generated using a reverse transcription premixed kit with gDNA Eraser (Accurate Biotechnology Co., Ltd., Changsha, China) according to the supplier’s protocol. RT-qPCR was carried out using SYBR Green Master Mix (Accurate Biotechnology Co., Ltd., Changsha, China) on a QuantStudio™ real-time PCR system (Applied Biosystems, Waltham, MA, USA). The amplification protocol included an initial denaturation step at 95 °C for 5 min, followed by 40 cycles of denaturation at 95 °C for 10 s, annealing at 60 °C for 20 s, and extension at 72 °C for 20 s. β-actin served as the internal reference gene. Relative mRNA expression levels of inducible nitric oxide synthase (Nos2), superoxide dismutase 2 (SOD2), CAT, and NAD(P)H quinone dehydrogenase 1 (Nqo1) were calculated using the 2^−ΔΔCT^ method. Primer sequences are provided in Table 1. Detailed procedures were performed as described in our previous study.

### 2.10. RNA Sequencing and Bioinformatic Analysis

Jejunal tissues were used for transcriptomic profiling. Total RNA was isolated using TRIzol reagent (Life Technologies, Carlsbad, CA, USA). RNA concentration and purity were evaluated using a NanoDrop 2000 spectrophotometer (Thermo Fisher Scientific, Waltham, MA, USA), while RNA integrity was assessed with an Agilent Bioanalyzer 2100 system (Agilent Technologies, Santa Clara, CA, USA). Library preparation and sequencing were carried out by Bio-Tree Biomedical Co., Ltd. (Shanghai, China). mRNA libraries were constructed from 1 μg of total RNA using the Hieff NGS Ultima Dual-mode mRNA Library Prep Kit (Yeasen Biotechnology, Shanghai, China). Poly(A)+ mRNA was enriched using oligo(dT) magnetic beads, followed by cDNA synthesis, adaptor ligation, and PCR amplification. The libraries were quality-checked and sequenced on the Illumina NovaSeq platform to generate 150 bp paired-end reads. Clean reads were aligned to the Sus scrofa reference genome (Sscrofa11.1, NCBI assembly) using HISAT2. Transcript assembly and quantification were performed using StringTie, and gene expression levels were normalized as FPKM (Fragments Per Kilobase of transcript per Million fragments mapped). Differential expression analysis between groups was performed using the DESeq2 package based on a negative binomial distribution model. Genes with an adjusted *p* value < 0.01 and |log2 fold change| ≥ 1 were defined as differentially expressed. Principal component analysis (PCA), sample correlation analysis, heatmap visualization, and module score calculation were conducted using normalized expression data to characterize global and pathway-associated transcriptional changes.

### 2.11. Statistical Analysis

All data are presented as mean ± standard deviation (SD). Statistical analysis was conducted using IBM SPSS Statistics 27.0 (IBM Corp., Armonk, NY, USA). Data normality was assessed using the Shapiro–Wilk test. Differences among three groups were analyzed by one-way analysis of variance (ANOVA) followed by Tukey’s post hoc test. Comparisons between two groups were performed using Student’s *t*-test. Transcriptomic data were analyzed separately using the DESeq2 package as described above. Graphs were plotted using GraphPad Prism 9.0 software (GraphPad Software, San Diego, CA, USA). Image processing and densitometric quantification of immunohistochemistry and Western blot bands were performed using ImageJ software (NIH, USA). A *p* value < 0.05 was considered statistically significant.

## 3. Results

### 3.1. YAM Confers Structural Protection to the Intestinal Mucosa

**Figure 1:**
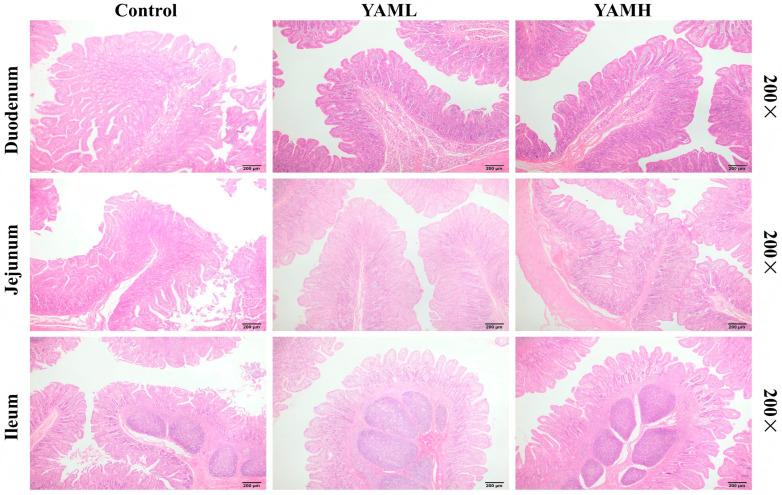
Effects of YAM supplementation on intestinal morphology in weaned piglets. Representative H&E-stained sections of the duodenum, jejunum, and ileum from the Control, YAML, and YAMH groups. YAML, low-dose YAM supplementation; YAMH, high-dose YAM supplementation. Scale bars = 200 μm (200× magnification).

H&E staining was performed to assess intestinal morphology. As shown in Figure 1, compared with the Control group, both YAML and YAMH groups showed improved villus architecture across all intestinal segments. The villi appeared more regularly arranged and elongated, with clearer epithelial borders and preserved structural integrity. In contrast, the Control group exhibited mild mucosal disruption and epithelial irregularities, whereas such features were less evident in the YAM-supplemented groups. No obvious villus breakage or epithelial shedding was observed in these groups. These observations suggest that dietary YAM may contribute to the maintenance of intestinal mucosal structure in weaned piglets. These histological findings are consistent with our previously published intestinal morphometric data [40].

### 3.2. YAM Improves Intestinal Antioxidant Status

The effects of YAM supplementation on intestinal antioxidant status are presented in Table 2. Compared with the Control group, dietary YAM significantly increased total antioxidant capacity (T-AOC) levels (*p* < 0.05). SOD activity was higher in the YAM-treated groups, although the difference was not statistically significant. No significant difference was observed in CAT activity between groups. MDA levels were lower in the YAM-treated groups, but the differences were not statistically significant. These results suggest that dietary YAM supplementation enhances intestinal antioxidant capacity in weaned piglets.

### 3.3. YAM Activates the Keap-1/Nrf2/HO-1 Antioxidant Signaling Pathway

**Figure 2:**
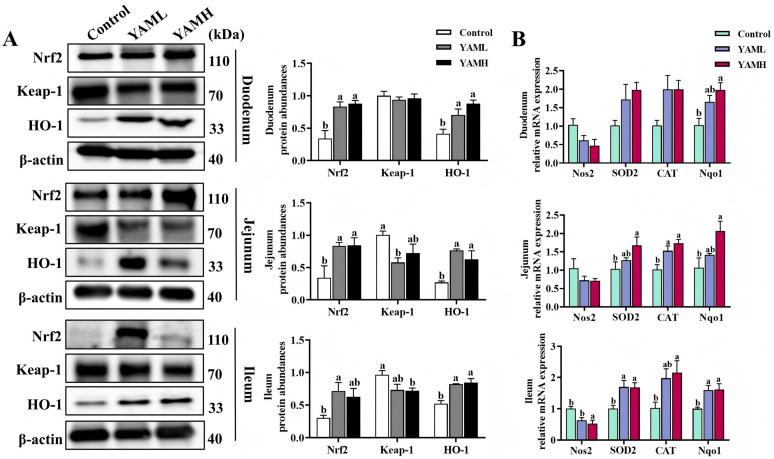
Effects of YAM on the intestinal Keap-1/Nrf2/HO-1 antioxidant signaling pathway in weaned piglets (*n* = 3). (**A**) Protein expression levels of Keap-1, Nrf2, and HO-1 in the duodenum, jejunum, and ileum were analyzed by Western blot. (**B**) Relative mRNA expression levels of antioxidant-related genes (Nos2, SOD2, CAT, and Nqo1) were determined by RT-qPCR. β-actin was used as the internal control. Different letters indicate significant differences (*p* < 0.05).

The activation of the intestinal Keap-1/Nrf2/HO-1 antioxidant pathway following YAM supplementation was evaluated by analyzing protein and gene expression levels. Protein expression analysis (Figure 2A) showed that, compared with the Control group, YAM supplementation enhanced Nrf2 antioxidant signaling. In the duodenum, both YAML and YAMH significantly increased Nrf2 and HO-1 protein expression (*p* < 0.05), while Keap-1 expression showed a decrease without statistical significance. In the jejunum, YAML and YAMH significantly upregulated Nrf2 and HO-1 protein levels (*p* < 0.05). Keap-1 expression was significantly reduced in the YAML group, whereas a downward trend was observed in the YAMH group. In the ileum, YAML significantly increased Nrf2 protein expression, and YAMH significantly decreased Keap-1 expression. Both YAML and YAMH markedly elevated HO-1 protein levels (*p* < 0.05). Gene expression analysis (Figure 2B) further supported activation of antioxidant responses. In the duodenum, no significant differences were observed in the mRNA expression of Nos2, SOD2, or CAT among the groups, whereas Nqo1 expression was significantly increased in the YAMH group. In the jejunum, Nos2 expression showed no significant difference among groups, whereas CAT and Nqo1 expression levels were significantly elevated, particularly in the YAMH group. In the ileum, both YAML and YAMH significantly decreased Nos2 expression and markedly increased SOD2, CAT, and Nqo1 mRNA levels (*p* < 0.05). Collectively, these findings indicate that YAM supplementation activates the Keap-1/Nrf2/HO-1 antioxidant pathway and upregulates downstream antioxidant genes in the intestine of weaned piglets.

### 3.4. YAM Attenuates Intestinal Inflammatory Signaling via NF-κB Inhibition

**Figure 3:**
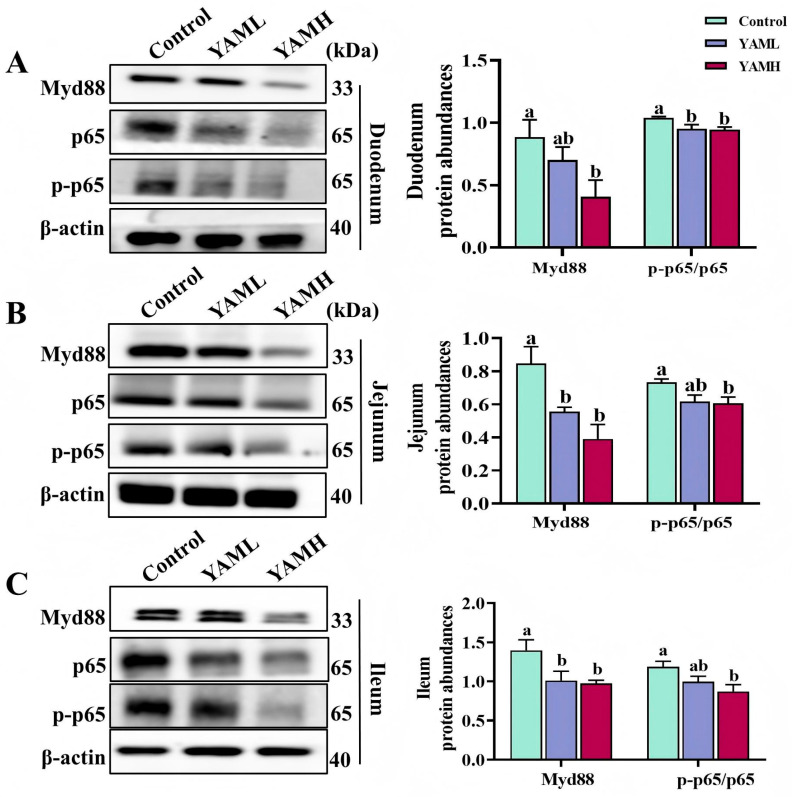
Effects of YAM supplementation on intestinal NF-κB signaling in weaned piglets. Protein expression of Myd88, p65, and p-p65 in the duodenum (**A**), jejunum (**B**), and ileum (**C**) was analyzed by Western blot. Representative bands and quantitative analyses are shown. Protein levels were normalized to β-actin. Different letters indicate significant differences (*p* < 0.05).

The effects of YAM supplementation on intestinal NF-κB signaling were evaluated by analyzing Myd88 and p-p65/p65 protein expression in different intestinal segments. Compared with the Control group, YAM supplementation markedly suppressed NF-κB pathway activation across the duodenum (Figure 3A), jejunum (Figure 3B), and ileum (Figure 3C). In the duodenum, the YAML group showed a significant reduction in p-p65/p65 expression, whereas the YAMH group significantly decreased both Myd88 and p-p65/p65 levels (*p* < 0.05). In the jejunum and ileum, YAML supplementation significantly downregulated Myd88 expression, while YAMH supplementation resulted in significant reductions in both Myd88 and p-p65/p65 protein levels (*p* < 0.05). These results indicate that dietary YAM supplementation suppresses activation of the NF-κB signaling pathway in the intestine of weaned piglets.

### 3.5. YAM Suppresses Mitochondrial-Mediated Epithelial Apoptosis

**Figure 4:**
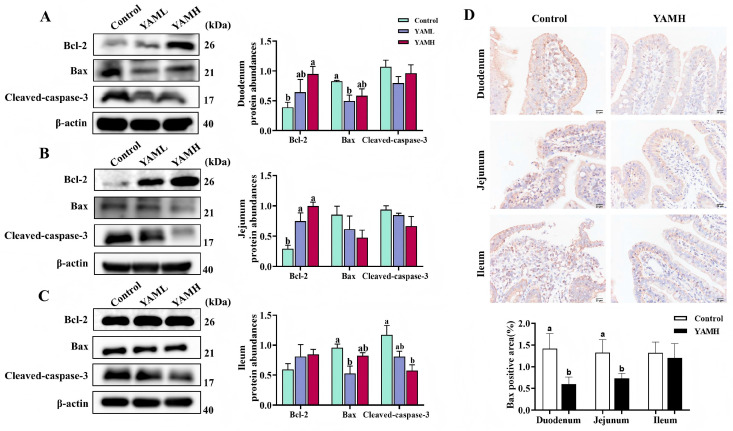
Effects of YAM on intestinal apoptosis in weaned piglets (*n* = 3). Protein expression levels of apoptosis-related markers (Bcl-2, Bax, and Cleaved caspase-3) in the duodenum (**A**), jejunum (**B**), and ileum (**C**) were analyzed by Western blot. (**D**) Immunohistochemical staining of Bax protein expression in different intestinal segments (200×, scale bar = 50 μm). Representative images from the Control and YAMH groups are shown. β-actin was used as the internal control. Different letters indicate significant differences (*p* < 0.05).

The effects of YAM supplementation on intestinal epithelial apoptosis were evaluated by analyzing apoptosis-related protein expression. In the duodenum (Figure 4A), the YAMH group significantly increased Bcl-2 expression (*p* < 0.05), while the YAML group significantly reduced Bax expression (*p* < 0.05). In the jejunum (Figure 4B), both YAML and YAMH supplementation significantly upregulated Bcl-2 protein expression (*p* < 0.05). No significant differences were observed in Bax or Cleaved caspase-3 levels among the groups. In the ileum (Figure 4C), no significant difference was observed in Bcl-2 expression among the groups, accompanied by a reduction in Cleaved caspase-3 expression. YAML significantly decreased Bax expression, whereas YAMH significantly reduced Cleaved caspase-3 levels. Immunohistochemical analysis further confirmed these findings (Figure 4D), showing reduced expression of the pro-apoptotic protein Bax following YAM supplementation. These results indicate that dietary YAM supplementation suppresses epithelial apoptosis in the intestine of weaned piglets.

### 3.6. Global Transcriptomic Regulation Induced by YAM in the Jejunum

#### 3.6.1. Differential Gene Expression Analysis

**Figure 5:**
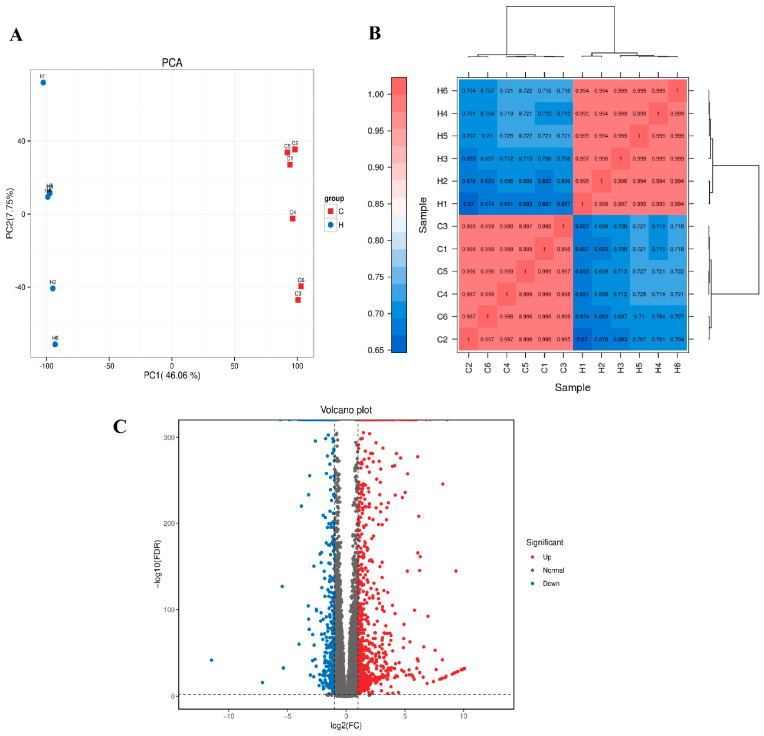
Global transcriptomic profiling of jejunal tissue in Control and YAMH groups. (**A**) Principal component analysis (PCA) based on normalized gene expression data showing clear separation between Control and YAMH groups. PC1 and PC2 explained 46.06% and 27.75% of the total variance, respectively. (**B**) Sample-to-sample Pearson correlation heatmap with hierarchical clustering, illustrating high within-group consistency and clear group segregation. (**C**) Volcano plot of differentially expressed genes (DEGs) between YAMH and Control groups. Red dots indicate upregulated genes, blue dots indicate downregulated genes, and gray dots represent non-significant genes (Normal). DEGs were defined according to the specified statistical thresholds.

To further characterize the transcriptional alterations underlying the protective effects of YAM supplementation, RNA sequencing was performed on jejunal tissues from the Control and YAMH groups. Principal component analysis (PCA) based on normalized transcriptomic data revealed a clear separation between the two groups (Figure 5A). PC1 accounted for 46.06% of the total variance, while PC2 explained 27.75%, together capturing 73.81% of the overall transcriptional variability. Samples within each group clustered closely and showed no overlap between groups, indicating distinct transcriptional profiles and strong intra-group consistency. To assess data reliability, sample-to-sample correlation analysis was conducted using Pearson correlation coefficients. Within-group correlations were consistently high (r = 0.97–0.99), whereas correlations between groups were comparatively lower (r = 0.68–0.75). Hierarchical clustering based on sample distance further separated Control and YAMH samples into two distinct clusters, supporting the stability of biological replicates and the absence of obvious outliers (Figure 5B). Differential expression analysis identified a total of 1227 differentially expressed genes (DEGs) between the two groups under the defined statistical criteria, including 784 upregulated genes and 443 downregulated genes in the YAMH group relative to the Control group. The volcano plot illustrated the overall distribution of DEGs, with a predominance of upregulated genes in response to YAM supplementation, indicating pronounced transcriptional changes in jejunal tissue (Figure 5C).

#### 3.6.2. Mechanism-Oriented Transcriptomic Signatures

**Figure 6:**
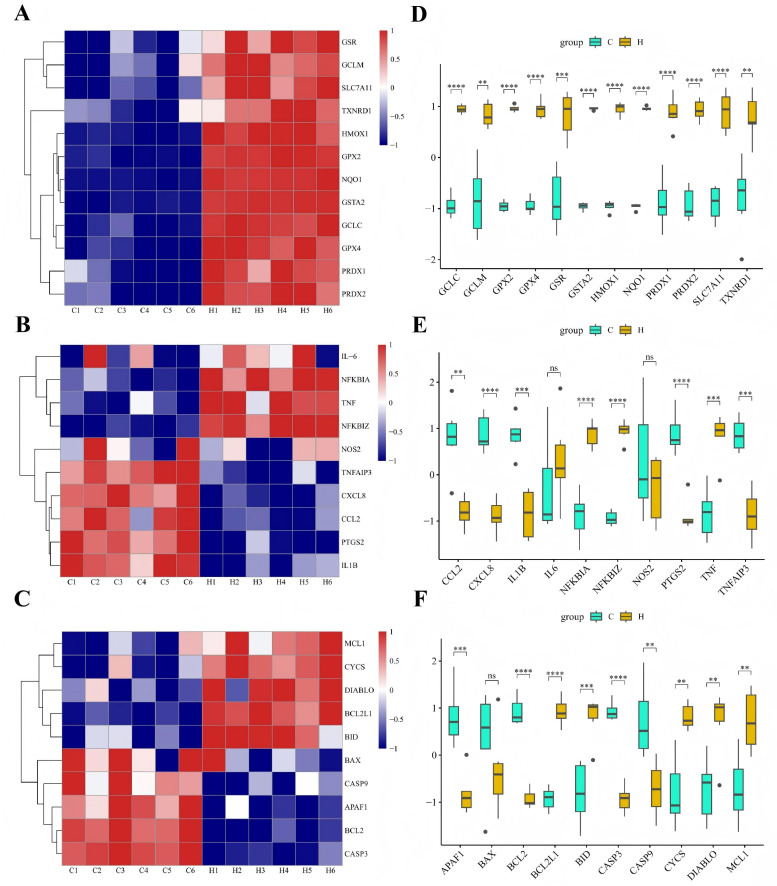
Mechanism-oriented transcriptomic signatures in jejunal tissue. (**A**–**C**) Heatmaps showing normalized expression profiles of genes associated with antioxidative defense (Nrf2-ARE targets), inflammatory output, and apoptosis-related pathways in the Control and YAMH groups. Expression values were transformed as log2(FPKM+1) and scaled by row Z-score for visualization. (**D**–**F**) Corresponding module scores calculated from the averaged log2(FPKM+1) expression values of representative genes within each functional category. Data are presented as mean ± SD (*n* = 6 per group). Statistical significance between groups was determined using an unpaired two-tailed Student’s *t*-test. ns = not significant, ** *p* < 0.01, *** *p* < 0.001, **** *p* < 0.0001.

To characterize transcriptomic changes that were consistent with the biochemical and protein-level findings, mechanism-oriented analyses were performed focusing on antioxidative defense, inflammatory output, and apoptosis-related gene expression. Heatmap visualization of representative Nrf2-ARE target genes showed coordinated upregulation in the YAMH group compared with the Control group (Figure 6A–C). Core antioxidant genes, including *HMOX1*, *NQO1*, *GPX2*, *GSTA2*, *GCLC*, and *SLC7A11*, exhibited consistently higher expression levels across YAMH samples, and row-scaled clustering clearly segregated the two groups. In contrast, genes associated with inflammatory mediators and downstream NF-κB transcriptional output, including *IL1B*, *PTGS2*, *CXCL8*, *CCL2*, and *NOS2*, displayed coordinated downregulation in the YAMH group. Analysis of apoptosis-related genes further revealed reduced expression of apoptosis-related genes. Expression levels of pro-apoptotic execution-related genes such as *CASP3*, *CASP9*, and *APAF1* were decreased in the YAMH group, while anti-apoptotic regulators showed no significant change or a slight increase. These patterns were clearly visualized in the heatmap and indicated consistent expression patterns across functional gene sets of antioxidant, inflammatory, and apoptotic pathways (Figure 6A–C). Consistent with these heatmap patterns, module score analysis further supported the coordinated regulation of these functional pathways (Figure 6D–F). The Nrf2-ARE module score was significantly increased in the YAMH group, whereas the inflammatory module score and apoptosis execution module score were significantly reduced. Together, these transcriptomic patterns were consistent with increased expression of antioxidant-related genes and decreased expression of inflammatory and apoptosis-related genes.

## 4. Discussion

Weaning is a critical period in piglets, during which abrupt dietary changes and intestinal immaturity often lead to oxidative stress, inflammation, and mucosal injury [41]. In this study, dietary YAM supplementation alleviated weaning-induced intestinal damage, as evidenced by improved mucosal morphology, reduced inflammatory signaling, enhanced antioxidant responses, and decreased epithelial apoptosis. The MyD88/NF-κB signaling pathway is a central regulator of inflammatory responses, in which MyD88 acts as a key adaptor protein mediating upstream signal transduction, while phosphorylation of p65 (p-p65/65) reflects activation of NF-κB and subsequent transcription of pro-inflammatory genes [42]. Transcriptomic analysis further supported these findings, indicating that YAM modulates oxidative stress and inflammation-related pathways in the jejunum. Collectively, these results suggest that YAM contributes to intestinal protection during the weaning transition.

Intestinal structural integrity is essential for nutrient digestion and absorption in weaned piglets, and weaning stress is frequently associated with villus damage and mucosal disruption [43,44]. In the present study, histological examination showed that YAM supplementation alleviated intestinal mucosal injury, as evidenced by more intact villus morphology and improved epithelial continuity. These findings suggest that YAM contributes to maintaining intestinal mucosal integrity during the weaning period, providing a morphological basis for its protective effects. These observations are consistent with our previously reported morphometric findings [40].

Inflammatory responses are recognized as a major contributor to intestinal injury during the weaning transition [45]. Activation of the NF-κB signaling pathway plays a central role in mediating mucosal inflammation, leading to epithelial damage and barrier dysfunction [46]. In the present study, YAM supplementation suppressed activation of the NF-κB pathway, as evidenced by reduced Myd88 expression and decreased p-p65/p65 levels in different intestinal segments. These findings suggest that the protective effects of YAM on intestinal structure are, at least in part, associated with the attenuation of inflammatory signaling. This effect may be related to the presence of bioactive constituents in YAM, particularly polysaccharides and steroidal saponins, which have been reported to exert anti-inflammatory activity through modulation of NF-κB signaling, reducing pro-inflammatory cytokine production (e.g., TNF-α and IL-6), and modulating oxidative stress responses, as described in previous studies [35], thereby contributing to the observed attenuation of intestinal inflammation and epithelial injury in the present study.

After weaning, the nutrient source of piglets shifts abruptly from milk to solid feed. Combined with intestinal immaturity, this transition disrupts redox balance and antioxidant defense systems, leading to excessive production of reactive ROS [43,47]. The resulting oxidative stress damages the intestinal mucosa and impairs barrier function [48]. In the present study, dietary supplementation with YAM improved antioxidant status in weaned piglets, as evidenced by increased T-AOC and reduced MDA concentrations. Although the activities of SOD and CAT did not change significantly, an overall trend toward enhanced antioxidant defenses was observed, suggesting activation of antioxidant responses in the intestinal mucosa. This finding is consistent with previous studies showing that YAM extracts alleviate oxidative injury by enhancing antioxidant defenses and suppressing inflammatory signaling [32]. In addition, diosgenin, a representative steroidal saponin from *Dioscorea* species, has been reported to activate Nrf2 signaling and limit intracellular ROS generation in both cellular and animal models [49]. Together, these findings provide a biochemical basis for the enhanced antioxidant capacity observed following YAM supplementation in the present study.

The Keap-1/Nrf2/HO-1 signaling pathway is a central regulator of cellular redox homeostasis, coordinating the transcription of antioxidant and phase II detoxifying enzymes [50]. In the present study, YAM supplementation promoted Nrf2 and HO-1 expression while reducing Keap-1 levels, accompanied by upregulation of downstream antioxidant genes, including CAT, SOD2, and Nqo1. These changes suggest that YAM may facilitate activation of the endogenous antioxidant defense system through modulation of redox-sensitive signaling pathways. YAM polysaccharides have been reported to enhance Nrf2 nuclear translocation and increase HO-1 expression in oxidative stress models, while diosgenin has been shown to modulate redox-sensitive pathways and attenuate ROS-mediated tissue injury [51,52]. These observations provide mechanistic support for the activation of the Keap-1/Nrf2/HO-1 axis observed in the present study and further support the role of YAM in maintaining intestinal redox balance during the weaning transition.

Oxidative stress is a major driver of epithelial apoptosis, and the dynamic balance between apoptosis and proliferation of intestinal epithelial cells is essential for maintaining intestinal homeostasis [53,54]. Weaning stress disrupts antioxidant capacity and intestinal stability, thereby accelerating epithelial apoptosis and contributing to barrier dysfunction [55,56]. Members of the Bcl-2 family are key regulators of mitochondrial apoptosis. The pro-apoptotic protein Bax promotes cell death, whereas the anti-apoptotic protein Bcl-2 counteracts this process by interacting with Bax and limiting downstream caspase-3 activation [57]. In the present study, dietary YAM supplementation increased Bcl-2 expression while reducing Bax and Cleaved caspase-3 levels in different intestinal segments. Immunohistochemical staining further confirmed decreased Bax expression in the intestinal mucosa. These findings suggest that YAM mitigates excessive epithelial apoptosis induced by weaning stress, thereby contributing to the preservation of intestinal barrier integrity and potentially alleviating inflammatory and oxidative injury. YAM polysaccharides have been reported to protect intestinal and hepatic tissues from oxidative injury by regulating mitochondrial apoptotic pathways, including modulation of Bcl-2 and Bax expression [58,59,60,61], thereby maintaining mitochondrial membrane stability, limiting cytochrome c release, and preventing downstream caspase activation. In addition, diosgenin has been shown to attenuate ROS-mediated apoptosis and inhibit caspase activation in experimental models [62]. Such evidence provides mechanistic support for the inhibition of epithelial apoptosis observed following YAM supplementation in the present study [63].

RNA-seq analysis provided further mechanistic insight into the intestinal protective effects of YAM. The results demonstrated that dietary supplementation with YAM not only improved intestinal integrity in weaned piglets but also modulated oxidative stress responses, inflammatory signaling, and apoptosis in the intestine. Consistent with these observations, transcriptomic analysis revealed coordinated regulation of genes associated with antioxidative defense, inflammatory signaling, and apoptosis. Specifically, representative Nrf2-ARE target genes, including *HMOX1*, *NQO1*, *GPX2*, and *GCLC*, were upregulated, whereas inflammatory mediators such as *IL1B*, *PTGS2*, *CXCL8*, and *NOS2* were downregulated. In addition, the expression of apoptosis-related genes, including *CASP3*, *CASP9*, and *APAF1*, was reduced. These transcriptional alterations were consistent with the biochemical and protein-level findings, suggesting that YAM may alleviate intestinal injury through coordinated modulation of antioxidant responses, inflammatory signaling pathways, and apoptotic processes.

## 5. Conclusions

In summary, the present study demonstrates that dietary YAM supplementation may effectively alleviate weaning-induced intestinal injury in piglets. The protective effects were characterized by improved mucosal morphology, suppression of NF-κB-mediated inflammatory signaling, enhancement of antioxidant capacity through activation of the Keap-1/Nrf2/HO-1 pathway, and inhibition of epithelial apoptosis. Transcriptomic profiling further supported these findings by revealing coordinated regulation of genes associated with antioxidative defense, inflammatory signaling, and apoptosis. These results indicate that YAM contributes to maintaining intestinal homeostasis during the weaning transition and may represent a promising nutritional strategy for improving gut health in weaned piglets.

## Figures and Tables

**Table 1:** Primer sequences used for RT-qPCR.

Gene	Forward Primer	Reverse (5′-3′)
Nos2	5′ -GGGTCAGAGCTACCATCCTC-3′	5′ -CGTCCATGCAGAGAACCTTG-3′
SOD2	5′ -GGCCTACGTGAACAACCTGA-3′	5′ -TGATTGATGTGGCCTCCACC-3′
CAT	5′ -ACATGGTCTGGGACTTCTGG-3′	5′ -CATGTGCCTGTGTCCATCTG-3′
Nqo1	5′ -GCTTACACATACGCTGCCAT-3′	5′ -GCCACAGAAATGCAAAGTGA-3′
β-actin	5′ -GGTCACCAGGGCTGCTTT-3′	5′ -ACTGTGCCGTTGACCTTGC-3′

**Table 2:** Effects of YAM supplementation on intestinal antioxidant parameters in weaned piglets.

Items	Control	YAML	YAMH	*p* Value
T-AOC (μmol/mL)	0.46 ± 0.01 ^b^	0.50 ± 0.01 ^a^	0.52 ± 0.01 ^a^	0.032
SOD (U/mL)	13.43 ± 1.34 ^b^	18.09 ± 1.91 ^ab^	19.57 ± 2.18 ^a^	0.093
MDA (nmol/mL)	57.50 ± 2.99 ^a^	48.14 ± 5.03 ^ab^	44.41 ± 2.21 ^b^	0.057
CAT (U/mL)	13.63 ± 1.75	27.14 ± 4.02	18.09 ± 6.04	0.111

Values are expressed as mean ± SD. Different superscript letters within the same row indicate significant differences among groups (*p* < 0.05). T-AOC, total antioxidant capacity; SOD, superoxide dismutase; MDA, malondialdehyde; CAT, catalase.

## Data Availability

The original contributions presented in this study are included in the article/Supplementary Materials. Further inquiries can be directed to the corresponding author.

## Supplementary Materials

The following supporting information can be downloaded at: https://www.mdpi.com/article/10.3390/vetsci13040365/s1. Table S1: Composition and nutrient levels of the basal diets (air-dry basis, %).
