# Supplementary material for: Dioscorea oppositifolia L. Attenuates Weaning-Induced Intestinal Injury by Regulating Oxidative Stress and Apoptosis in Piglets
Source: Vet Sci. 2026 Apr 8;13(4):365. doi: 10.3390/vetsci13040365 (PMC13119762; doi:10.3390/vetsci13040365)
Supplement: Supplementary file 1 [file vetsci-13-00365-s001.zip › vetsci-4224728-raw data/Figure 2A.pptx]

## Slide 1
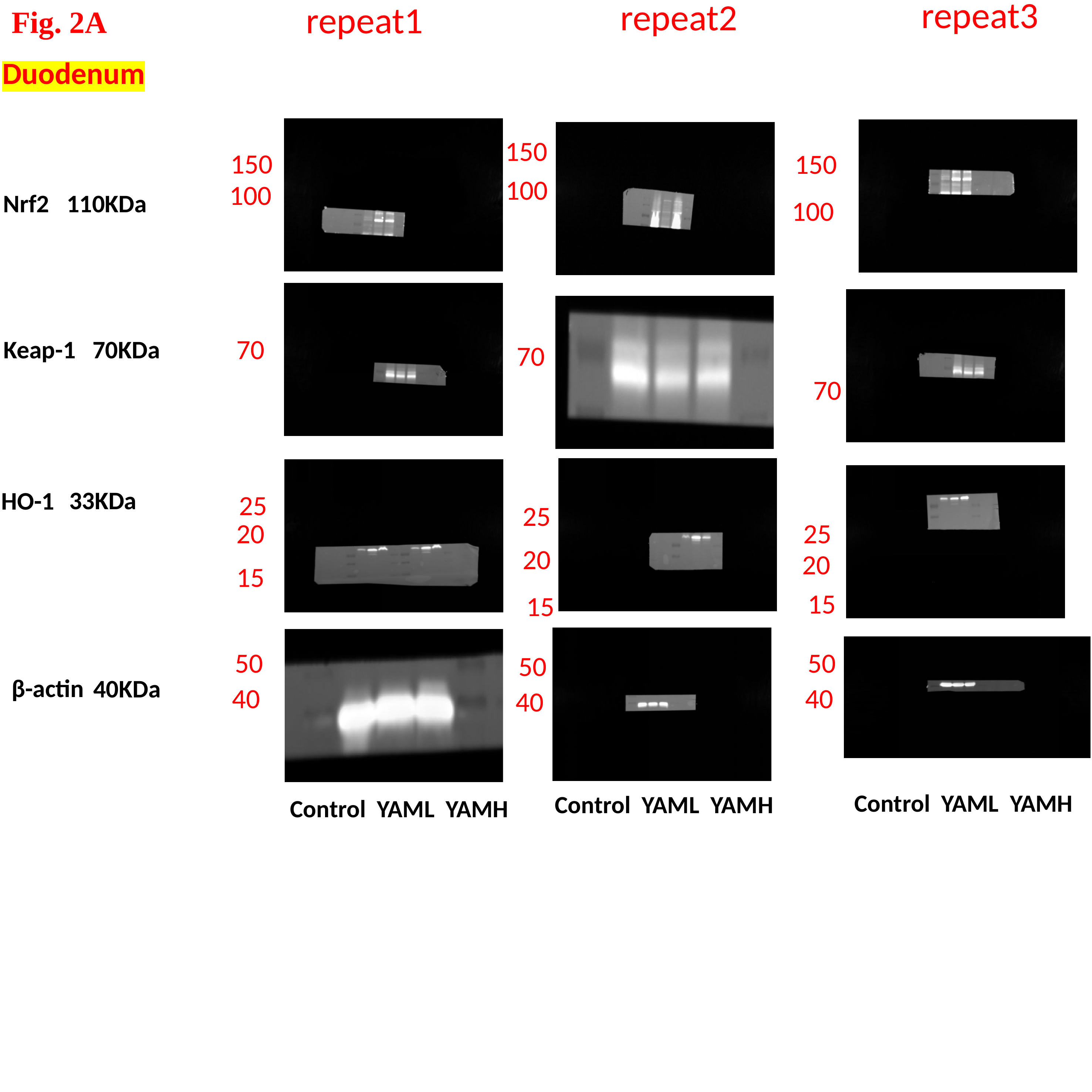

repeat3
repeat2
repeat1
Fig. 2A
Duodenum
150
100
150
100
150
100
Nrf2
110KDa
70
Keap-1
70KDa
70
70
33KDa
HO-1
25
20
15
25
20
15
25
20
15
50
40
50
40
50
40
β-actin
40KDa
Control YAML YAMH
Control YAML YAMH
Control YAML YAMH

## Slide 2
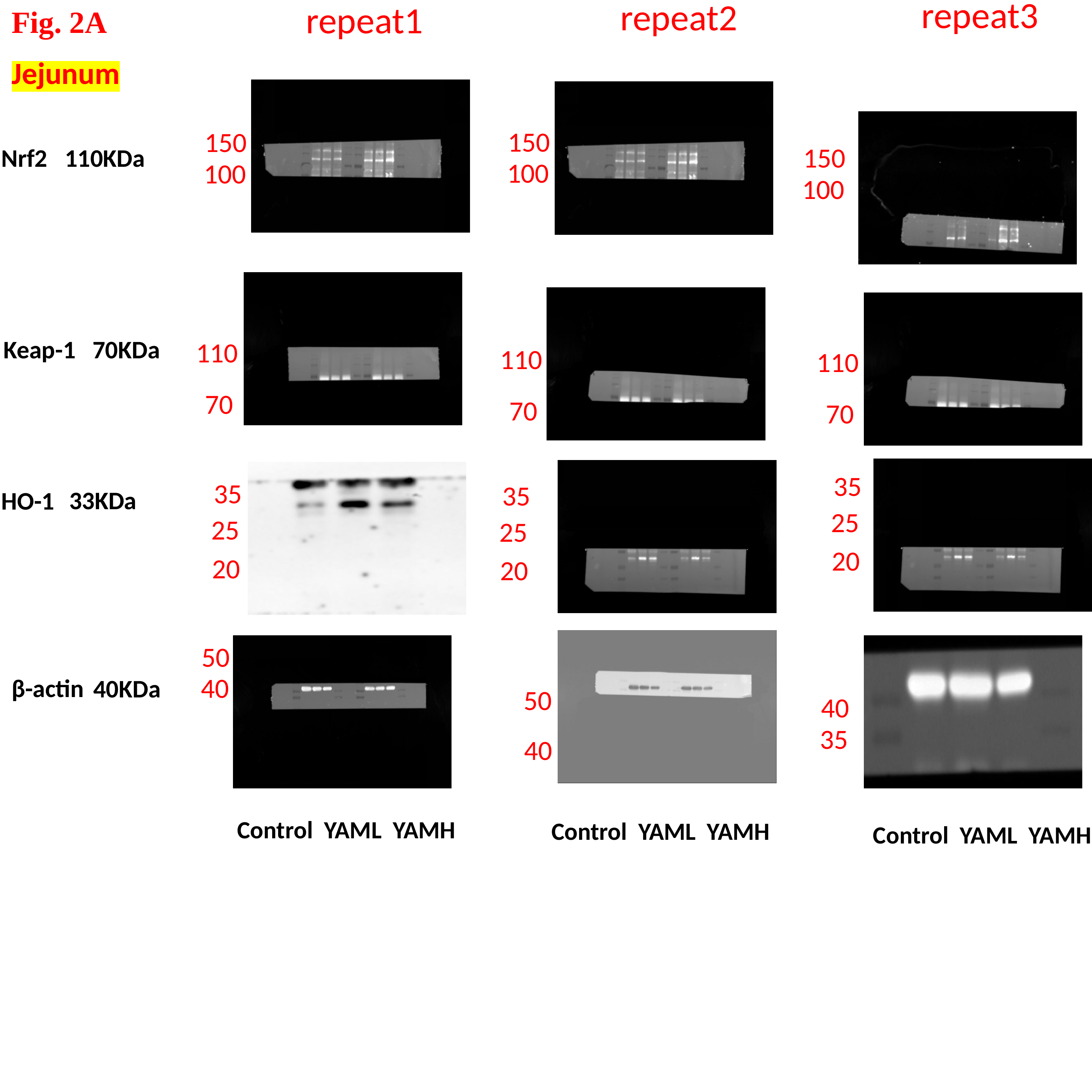

repeat3
repeat2
repeat1
Fig. 2A
Jejunum
150
100
150
100
150
100
Nrf2
110KDa
Keap-1
70KDa
110
70
110
70
110
70
35
25
20
35
25
20
35
25
20
33KDa
HO-1
50
40
β-actin
40KDa
50
40
40
35
Control YAML YAMH
Control YAML YAMH
Control YAML YAMH

## Slide 3
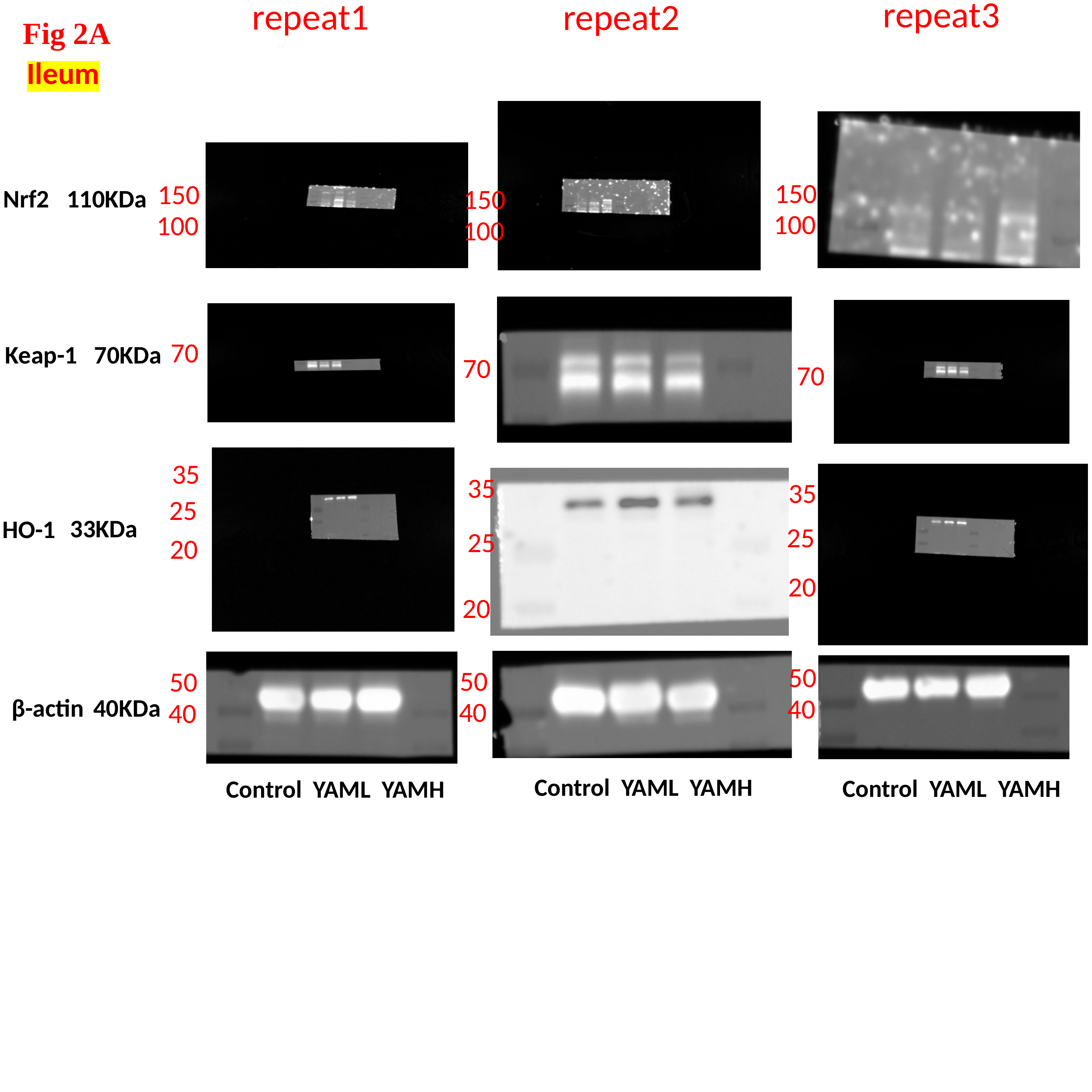

repeat3
repeat1
repeat2
Fig 2A
Ileum
150
100
150
100
150
100
Nrf2
110KDa
70
70
70
Keap-1
70KDa
35
25
20
35
25
20
35
25
20
33KDa
HO-1
50
40
50
40
50
40
β-actin
40KDa
Control YAML YAMH
Control YAML YAMH
Control YAML YAMH
