# Supplementary material for: Dioscorea oppositifolia L. Attenuates Weaning-Induced Intestinal Injury by Regulating Oxidative Stress and Apoptosis in Piglets
Source: Vet Sci. 2026 Apr 8;13(4):365. doi: 10.3390/vetsci13040365 (PMC13119762; doi:10.3390/vetsci13040365)
Supplement: Supplementary file 1 [file vetsci-13-00365-s001.zip › vetsci-4224728-raw data/Figure 3A-C.pptx]

## Slide 1
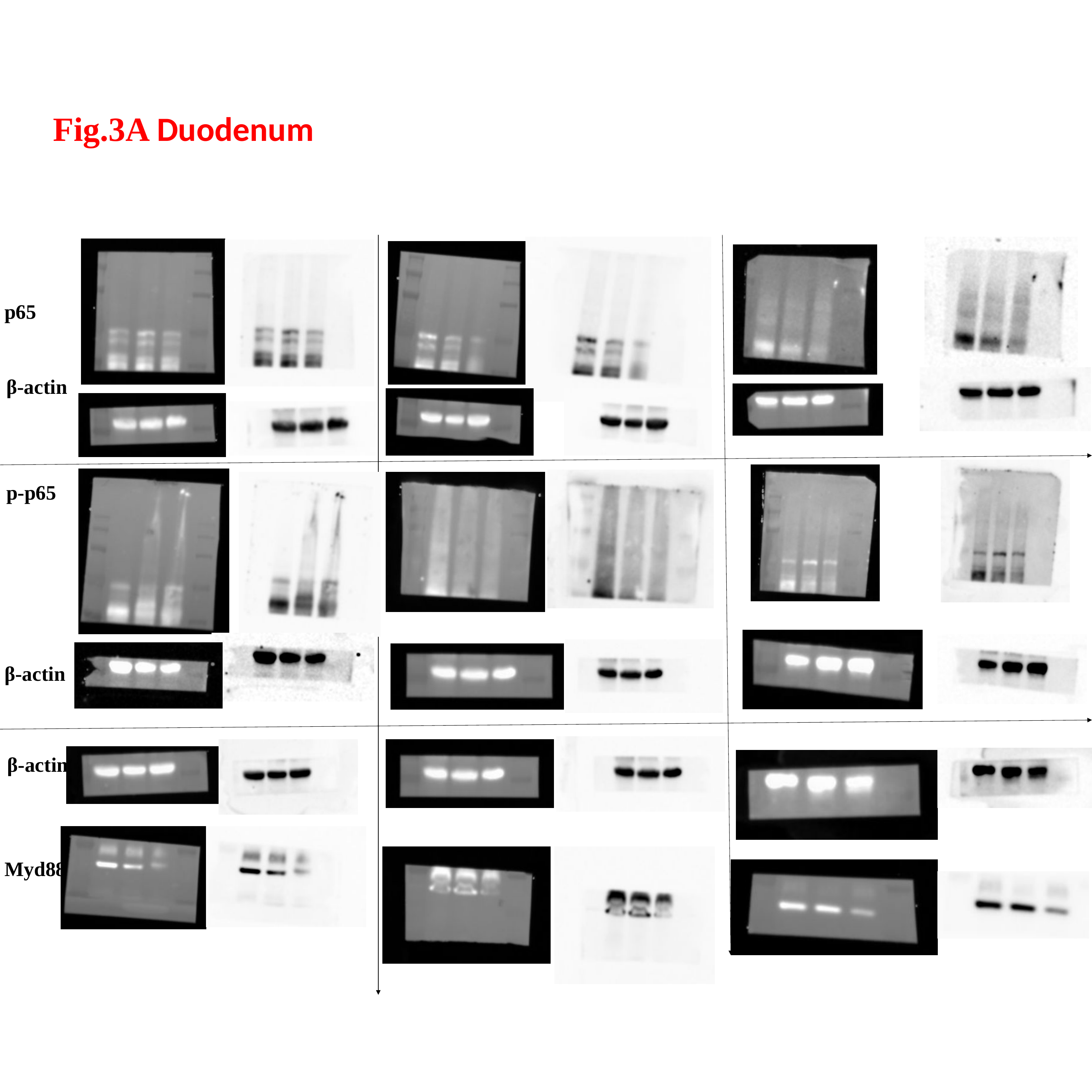

Fig.3A Duodenum
p65
β-actin
p-p65
β-actin
β-actin
Myd88

## Slide 2
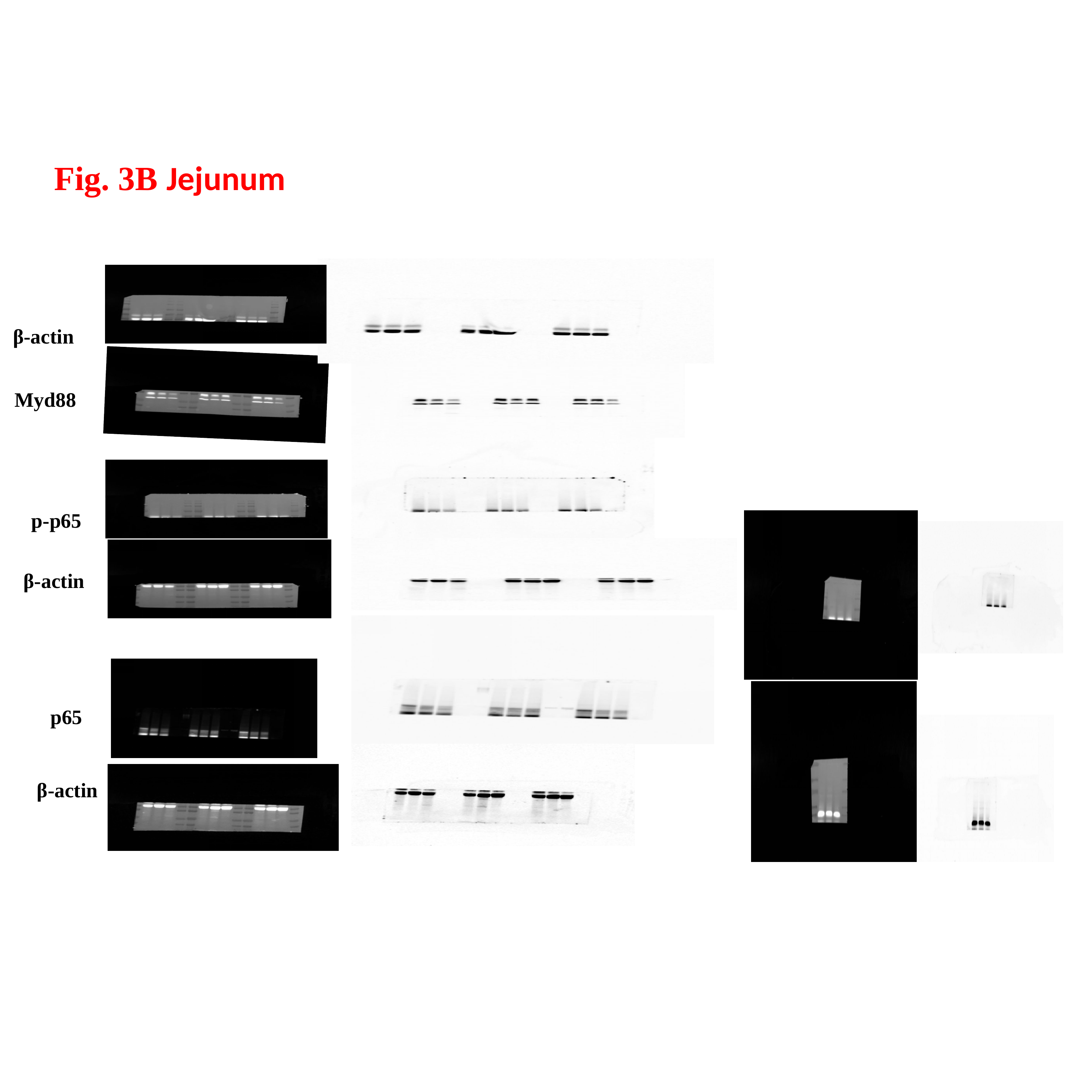

β-actin
Myd88
p-p65
β-actin
p65
β-actin
Fig. 3B Jejunum

## Slide 3
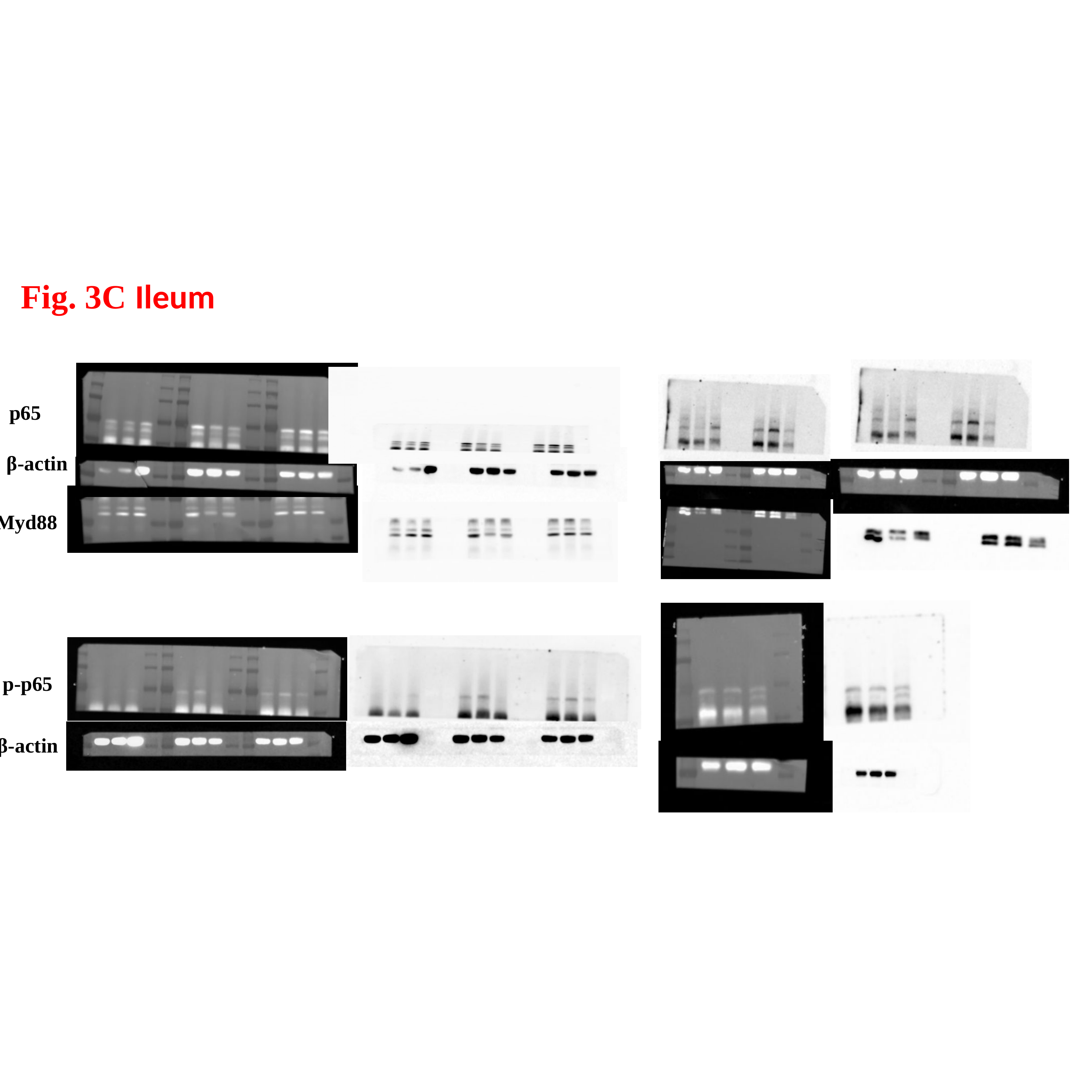

Fig. 3C Ileum
p65
β-actin
Myd88
p-p65
β-actin
