# Supplementary material for: Dioscorea oppositifolia L. Attenuates Weaning-Induced Intestinal Injury by Regulating Oxidative Stress and Apoptosis in Piglets
Source: Vet Sci. 2026 Apr 8;13(4):365. doi: 10.3390/vetsci13040365 (PMC13119762; doi:10.3390/vetsci13040365)
Supplement: Supplementary file 1 [file vetsci-13-00365-s001.zip › vetsci-4224728-raw data/Figure 4A-C.pptx]

## Slide 1
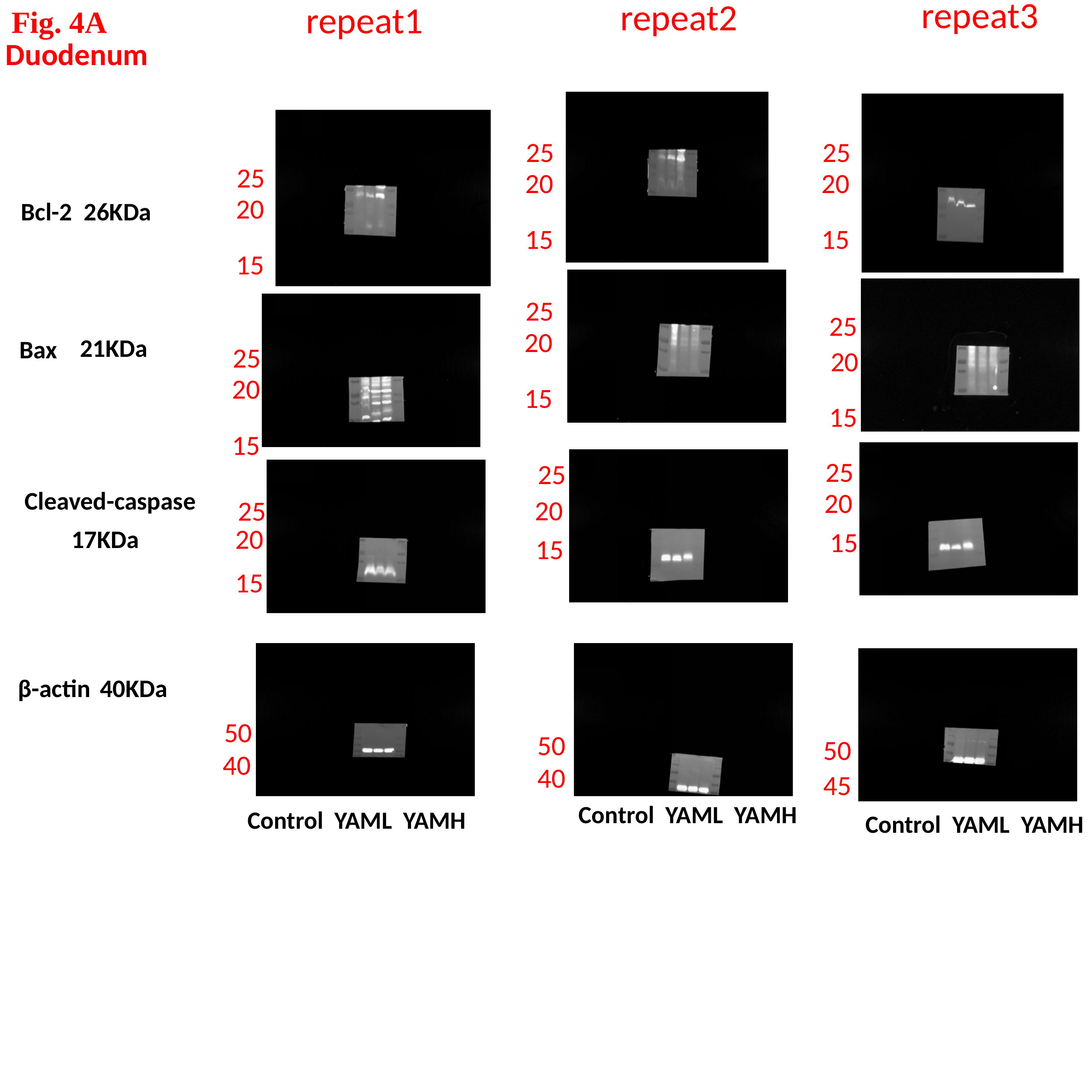

repeat3
repeat2
repeat1
Fig. 4A
Duodenum
25
20
15
25
20
15
25
20
15
Bcl-2
26KDa
25
20
15
25
20
21KDa
Bax
25
20
15
15
25
20
15
25
20
15
Cleaved-caspase
17KDa
25
20
15
β-actin
40KDa
50
40
50
40
50
45
Control YAML YAMH
Control YAML YAMH
Control YAML YAMH

## Slide 2
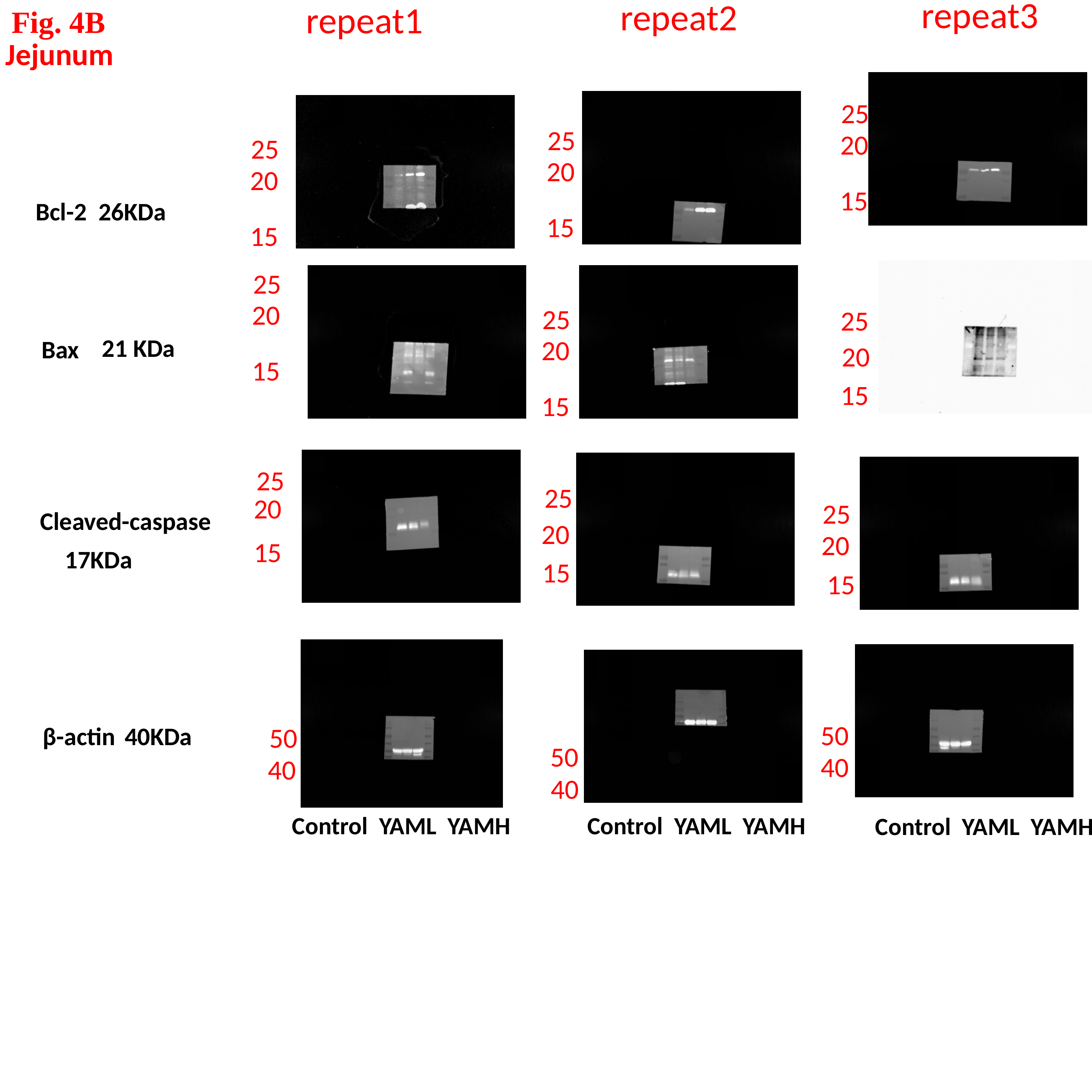

repeat3
repeat2
repeat1
Fig. 4B
Jejunum
25
20
15
25
20
15
25
20
15
Bcl-2
26KDa
25
20
15
25
20
15
25
20
21 KDa
Bax
15
25
20
15
25
20
15
25
20
15
Cleaved-caspase
17KDa
50
40
β-actin
40KDa
50
40
50
40
Control YAML YAMH
Control YAML YAMH
Control YAML YAMH

## Slide 3
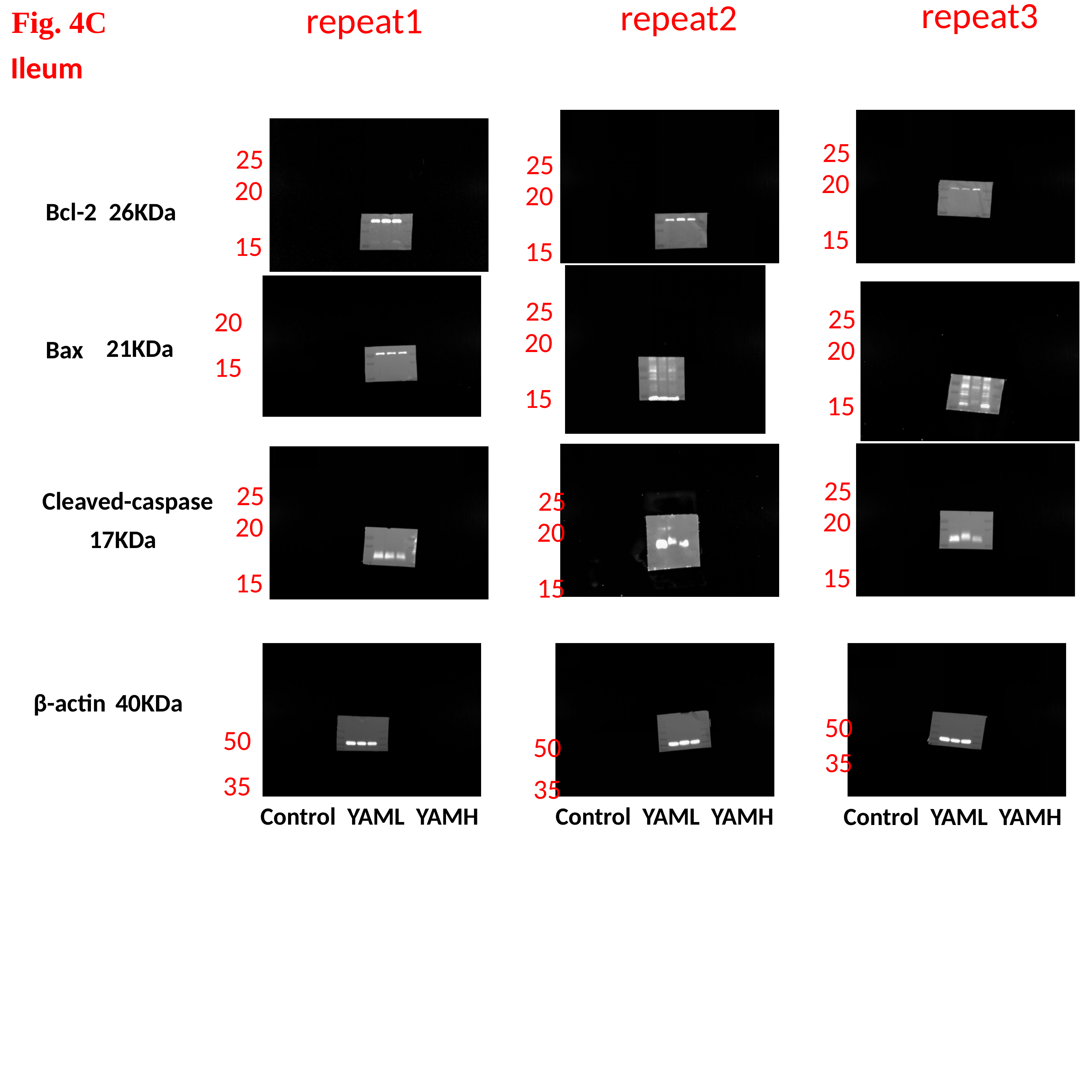

repeat3
repeat2
repeat1
Fig. 4C
Ileum
25
20
15
25
20
15
25
20
15
Bcl-2
26KDa
20
15
25
20
15
25
20
15
21KDa
Bax
25
20
15
25
20
15
25
20
15
Cleaved-caspase
17KDa
β-actin
40KDa
50
35
50
35
50
35
Control YAML YAMH
Control YAML YAMH
Control YAML YAMH
