# Supplementary material for: Dioscorea oppositifolia L. Attenuates Weaning-Induced Intestinal Injury by Regulating Oxidative Stress and Apoptosis in Piglets
Source: Vet Sci. 2026 Apr 8;13(4):365. doi: 10.3390/vetsci13040365 (PMC13119762; doi:10.3390/vetsci13040365)
Supplement: Supplementary file 1 [file vetsci-13-00365-s001.zip › vetsci-4224728-supplementary-major.pdf]

Table 1. Composition and nutrient levels of the basal diets (air-dry basis, %)

Ingredients	Content	Nutrient level[s]	Content
Corn	28.71	Digestion energy(Kcal/Kg)	3446.24
Broken rice	20.00	Crude protein	17.00
Soybean-puffed	8.00	Crude fiber	2.58
Soybean meal	14.88	Calcium	0.69
Glucose	2.00	Total phosphorus	0.49
fish meal	2.00	Total lysine	1.33
soy sauce	0.39	Total methionine	0.44
White sugar	2.0	Total threonine	0.86
Whey powder	3.0	Total Sulfur Amino acids	0.72
Whole milk treasure	5.0	Total Tryptophan	0.19
L-lysine hydrochloride	0.35	Total Isoleucine	0.70
Methionine	0.15	Total Valine	0.80
L-threonine	0.23		
High-temperature resistant phytase	0.03		
Complex enzymes	0.05		
Kejian peptide	0.20		
Wheat	10.00		
3% nursery feed premix [a]	3.00		
Total	100		

Note: [a]Premix provided per kilogram of basal diet: 9555 IU vitamin A, 3000 IU vitamin D3, 81 IU vitamin E, 3 mg vitamin K3, 3 mg vitamin B, 10.2 mg vitamin B2, 6 mg vitamin B6, 0.036 mg vitamin B12, 0.24 mg D-biotin, 30 mg D-pantothenic acid, 36 mg niacinamide, 300.06 mg choline chloride, 135 mg Fe, 115 mg Cu, 60 mg Mn, 1144.5 mg Zn, 118.2 mg methionine, 1638 mg L-lysine.

[b]. Nutrient levels were calculated values.
