# Supplementary figures and images for: Dioscorea oppositifolia L. Attenuates Weaning-Induced Intestinal Injury by Regulating Oxidative Stress and Apoptosis in Piglets
Source: Vet Sci. 2026 Apr 8;13(4):365. doi: 10.3390/vetsci13040365 (PMC13119762; doi:10.3390/vetsci13040365)

## Slide 1
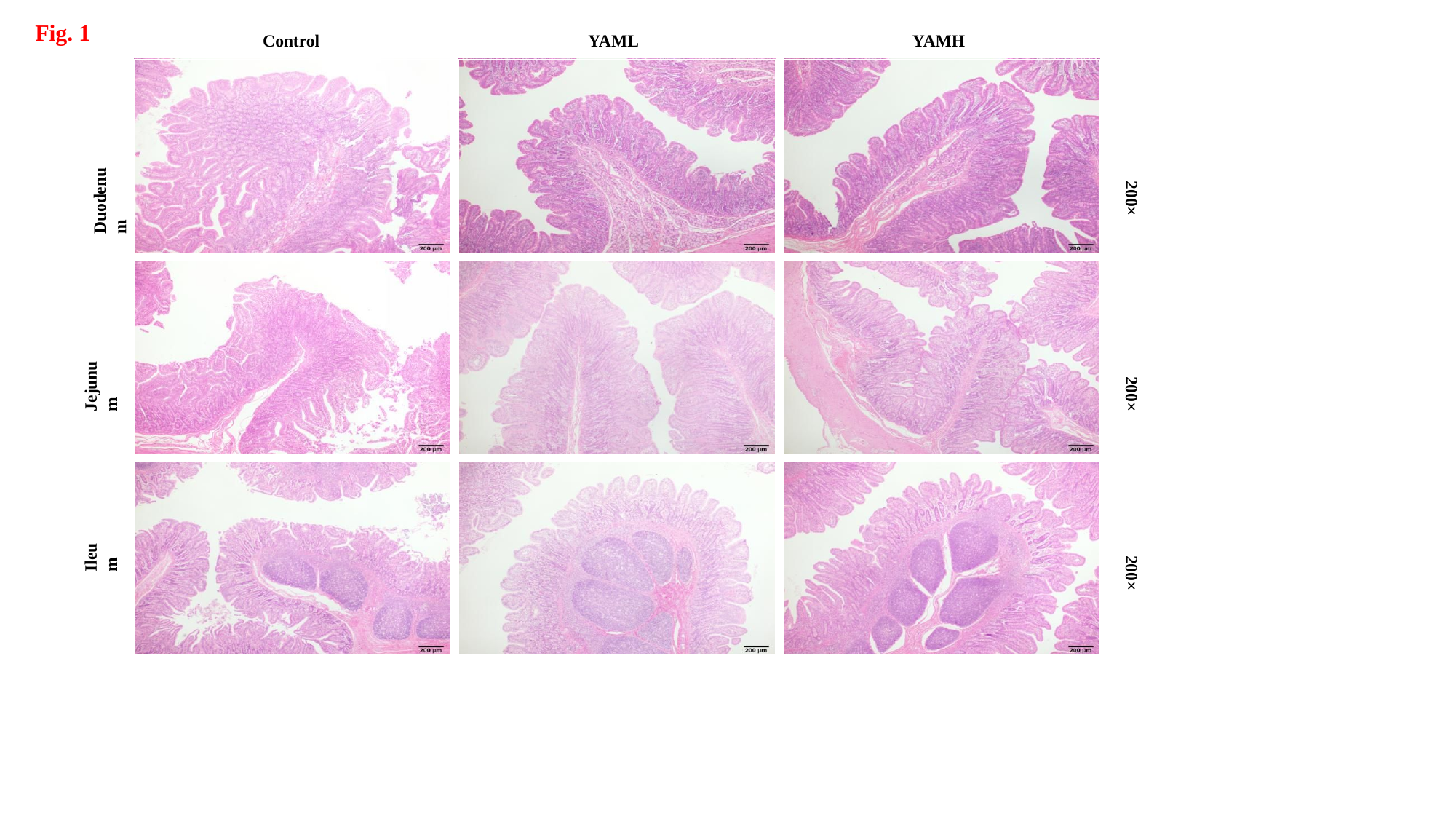

Fig. 1
Control
YAML
YAMH
200×
Duodenum
Jejunum
200×
Ileum
200×

Supplement: Supplementary file 1 [file vetsci-13-00365-s001.zip › vetsci-4224728-raw data/Figure 1.pptx]

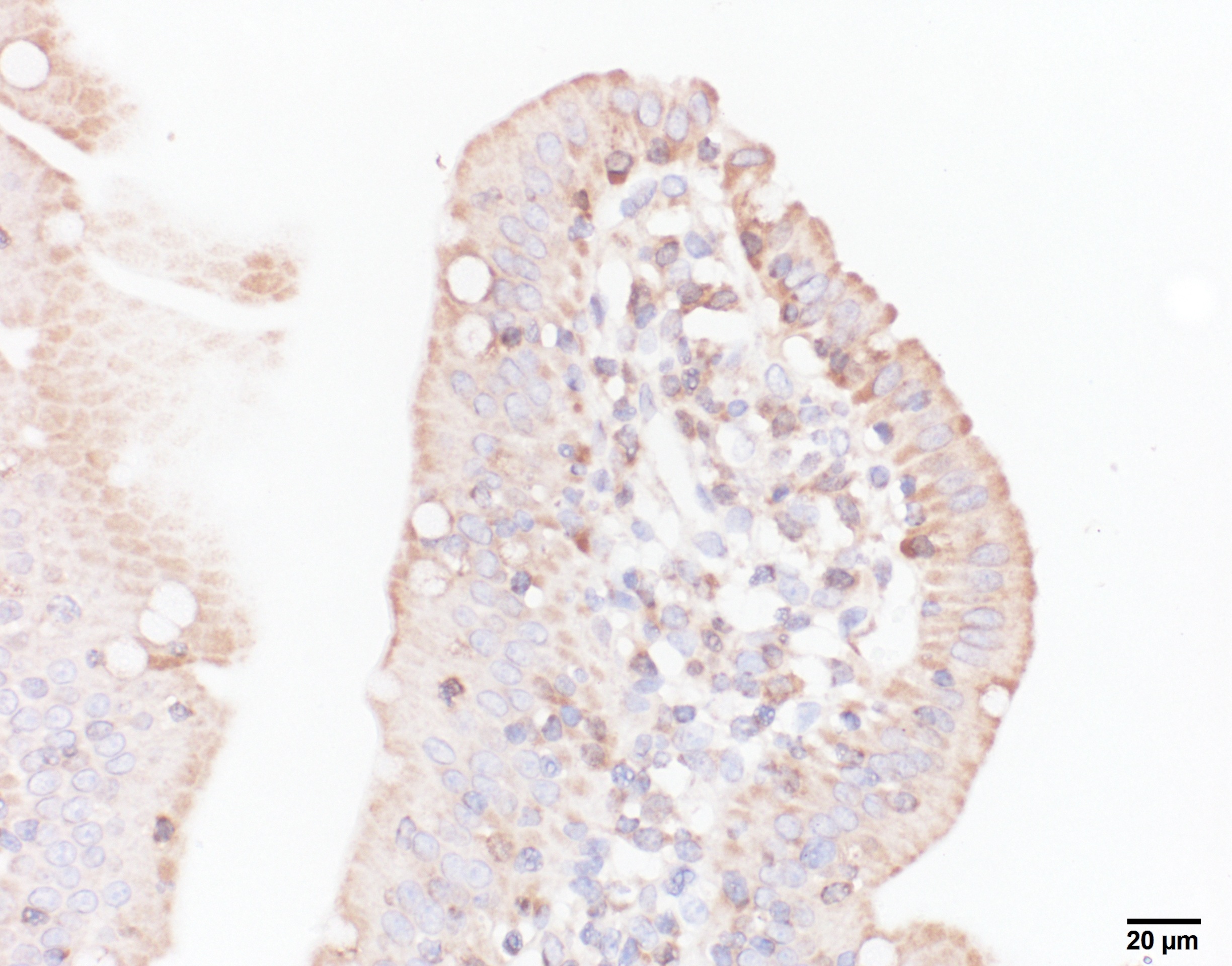

Supplement: Supplementary file 1 [file vetsci-13-00365-s001.zip › vetsci-4224728-raw data/Figure 4D/duodenum/C3.jpg]

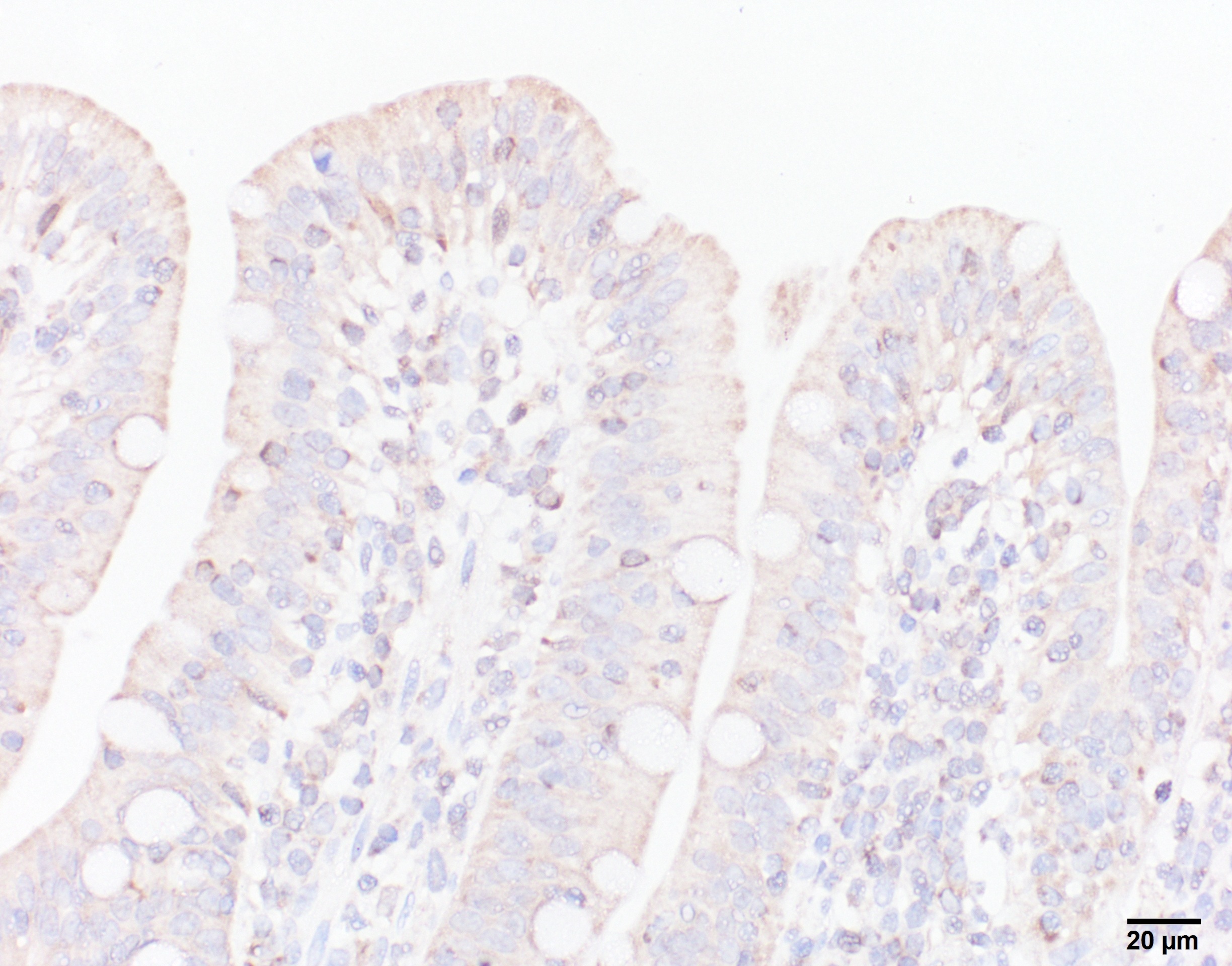

Supplement: Supplementary file 1 [file vetsci-13-00365-s001.zip › vetsci-4224728-raw data/Figure 4D/duodenum/H2.jpg]

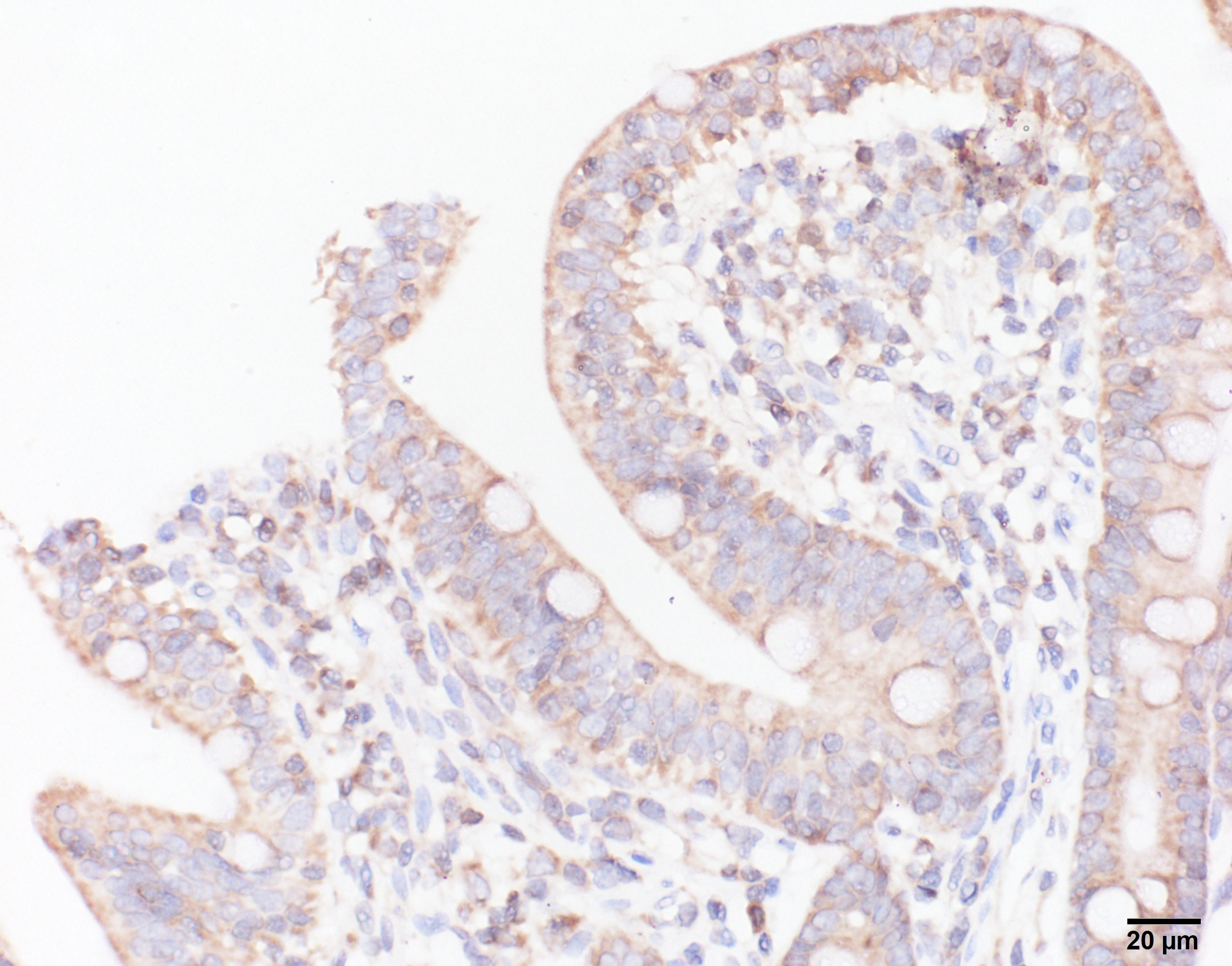

Supplement: Supplementary file 1 [file vetsci-13-00365-s001.zip › vetsci-4224728-raw data/Figure 4D/ileum/C1.jpg]

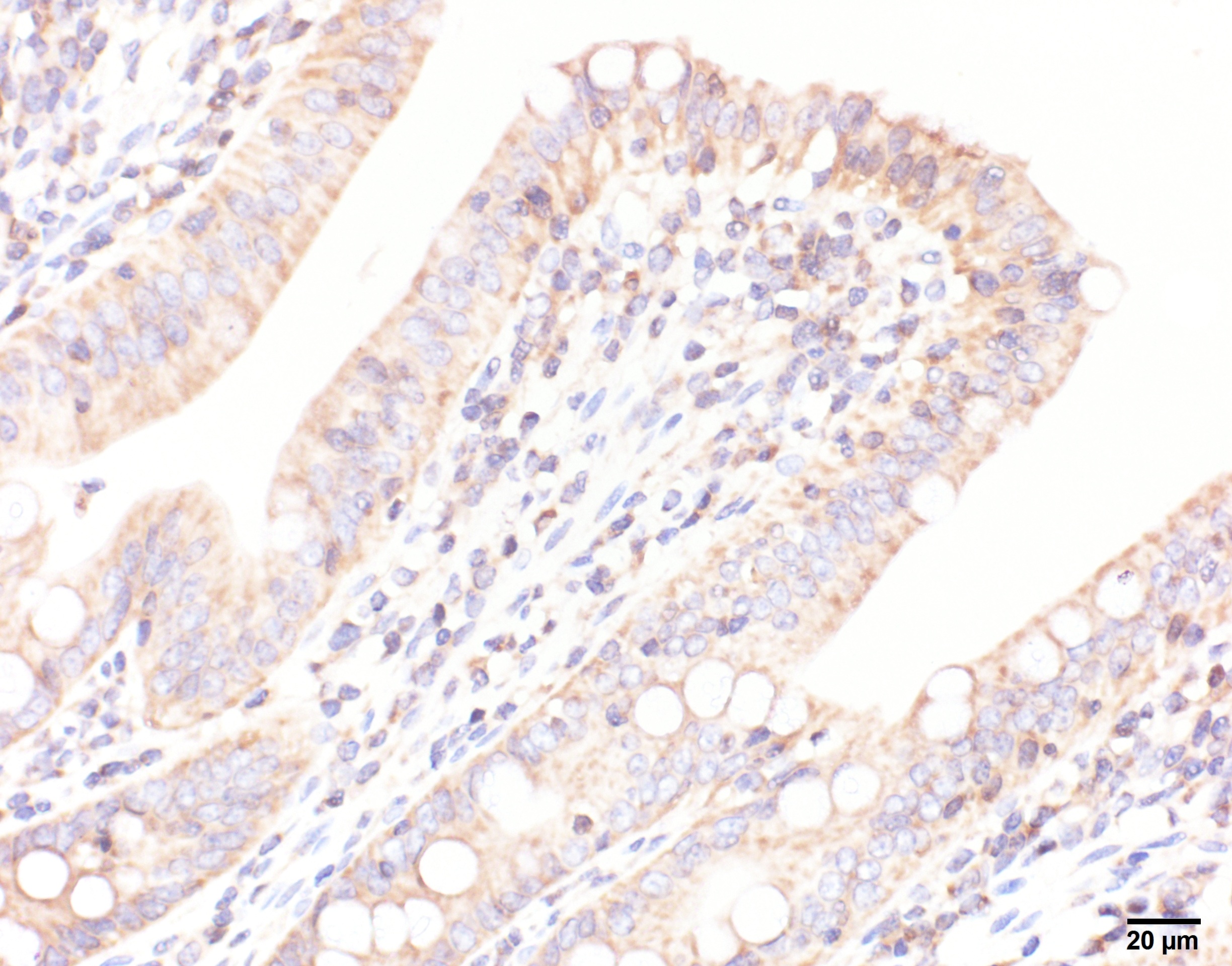

Supplement: Supplementary file 1 [file vetsci-13-00365-s001.zip › vetsci-4224728-raw data/Figure 4D/ileum/H2.jpg]

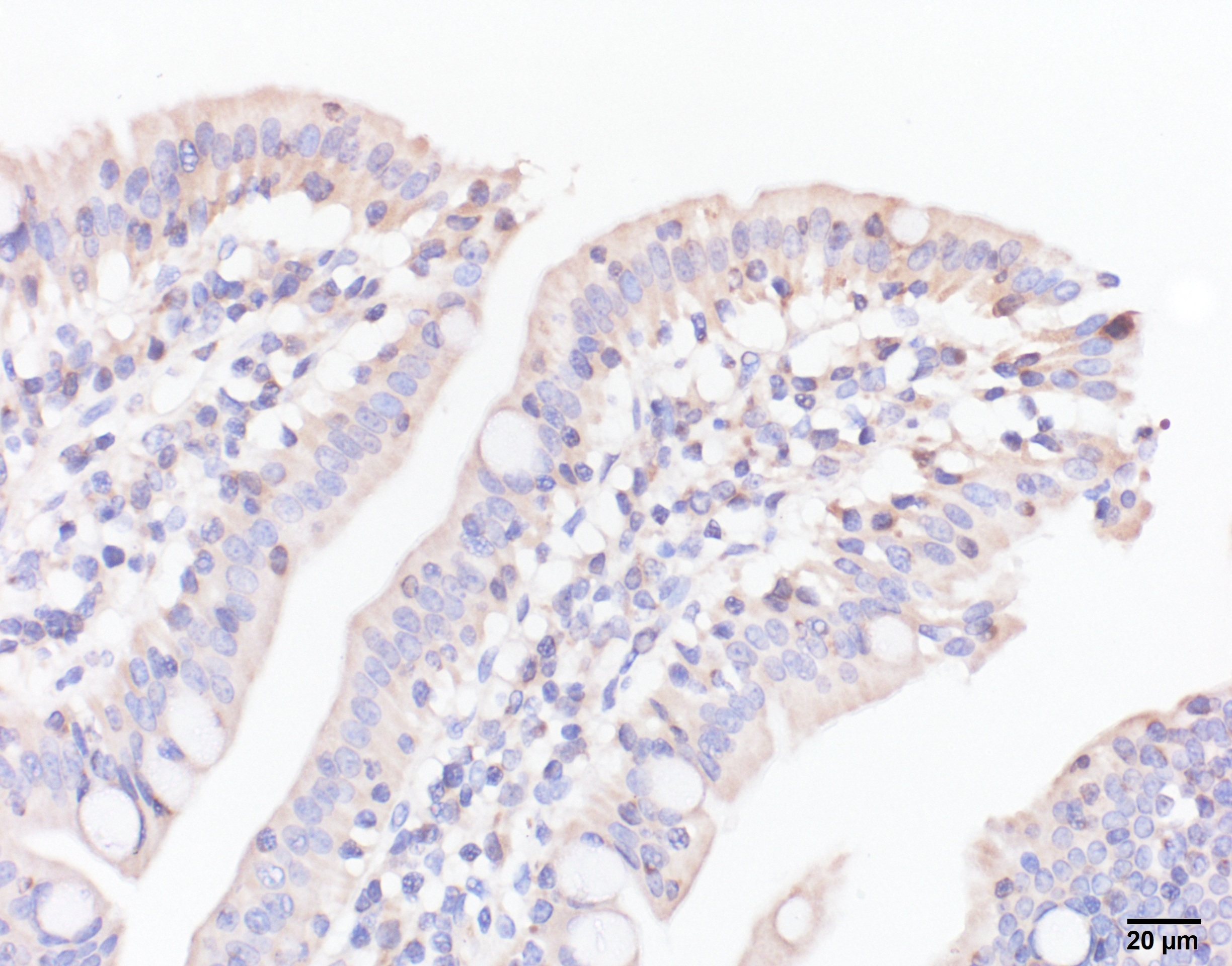

Supplement: Supplementary file 1 [file vetsci-13-00365-s001.zip › vetsci-4224728-raw data/Figure 4D/jejunum/C2.jpg]

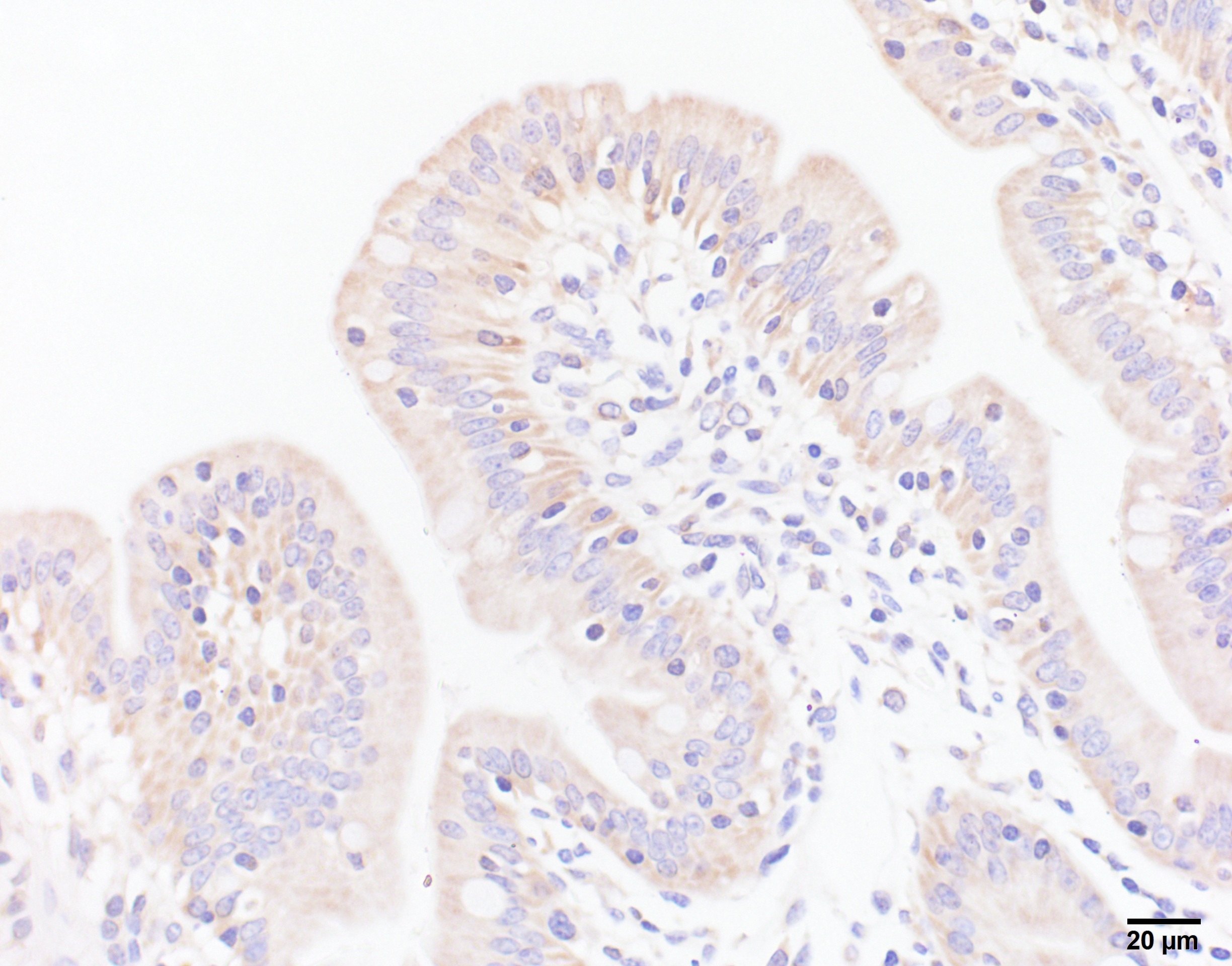

Supplement: Supplementary file 1 [file vetsci-13-00365-s001.zip › vetsci-4224728-raw data/Figure 4D/jejunum/H1.jpg]
